# The Choroid Plexus Functions as a Niche for T-Cell Stimulation Within the Central Nervous System

**DOI:** 10.3389/fimmu.2018.01066

**Published:** 2018-05-16

**Authors:** Itai Strominger, Yehezqel Elyahu, Omer Berner, Jensen Reckhow, Kritika Mittal, Anna Nemirovsky, Alon Monsonego

**Affiliations:** ^1^The Shraga Segal Department of Microbiology, Immunology and Genetics, Faculty of Health Sciences, Ben-Gurion University of the Negev, Beer Sheva, Israel; ^2^Zlotowski Center for Neuroscience, Ben-Gurion University of the Negev, Beer Sheva, Israel; ^3^The National Institute of Biotechnology in the Negev, Ben-Gurion University of the Negev, Beer Sheva, Israel

**Keywords:** central nervous system, choroid plexus, cerebrospinal fluid, CD4 T cell, antigen-presenting cell, migration, neuroinflammation

## Abstract

The choroid plexus (CP) compartment in the ventricles of the brain comprises fenestrated vasculature and, therefore, it is permeable to blood-borne mediators of inflammation. Here, we explored whether T-cell activation in the CP plays a role in regulating central nervous system (CNS) inflammation. We show that CD4 T cells injected into the lateral ventricles adhere to the CP, transmigrate across its epithelium, and undergo antigen-specific activation and proliferation. This process is enhanced following peripheral immune stimulation and significantly impacts the immune signaling induced by the CP. *Ex vivo* studies demonstrate that T-cell harboring the CP through its apical surface is a chemokine- and adhesion molecule-dependent process. We suggest that, within the CNS, the CP serves an immunological niche, which rapidly responds to peripheral inflammation and, thereby, promotes two-way T-cell trafficking that impact adaptive immunity in the CNS.

## Introduction

The choroid plexus (CP)—a compartment within the ventricles of the brain—manufactures most of the cerebrospinal fluid (CSF) (1) and serves as an interface between the blood and the central nervous system (CNS). The CP primarily comprises a fenestrated vasculature, a stroma, and epithelial cells, whose apical surfaces face the CSF (2). The CP has been implicated as a potential site for immune interactions between the immune system and the CNS, as it is known to house various resident immune cells (3–5), including CD4 T cells, macrophages (known as epiplexus cells or Kolmer cells), and CD11c^+^ dendritic cells (DCs) (2, 5–8). Although these cells may play essential roles in regulating cell-mediated immunity within the CNS, key questions regarding their trafficking to and across the CP as well as their role in regulating CNS inflammation remain unanswered.

The epithelial cells of the CP serve as a blood–CSF barrier, which determines the degree to which certain molecules and cells can translocate from the blood to the CSF. These epithelial cells express chemokines (such as CXCL10 and CCL20) under normal conditions (9, 10), and presumably participate in the upregulation of chemokines following a peripheral stimulus *in vivo* (11, 12). Such upregulation of chemokines by CP epithelial cells was also observed following stimulation with tumor necrosis factor (TNF) or interferon gamma (IFN-γ) *in vitro* (13). On their apical (CSF-facing) side, CP epithelial cells express adhesion molecules, such as the intercellular adhesion molecule 1 (ICAM-1) and the vascular cell adhesion molecule 1 (VCAM-1) (2, 14), which are upregulated following immune stimulation (13, 15, 16). The expression of chemokines and adhesion molecules on the apical surface of the CP epithelium may facilitate the homing of leukocytes from the CSF to the CP, thus facilitating their interaction with the CP epithelium and with local antigen-presenting cells (APCs). Such interactions may serve to modulate and amplify the immune milieu of the CP and thus its gateway functions within the CNS. However, to date, this function has only been speculated upon (4, 5, 17).

The CSF contains CD4 T cells, which exhibit primarily memory phenotypes, both in healthy individuals and in patients with neurological symptoms (18–20). For instance, in mice, T cells have been found in the CP under both healthy (13, 21, 22) and neuroinflammatory conditions, such as experimental autoimmune encephalomyelitis (10, 23). These and other studies suggest that CD4 T cells migrate from the blood to the CSF by crossing either the CP epithelium (10, 23, 24) or the meningeal vasculature (24–26). Here, we examine the role of the CP compartment in promoting the homing and activation of CD4 T cells, as a pathway that may precondition the CNS to immune surveillance.

## Results

### Innate Immune Stimulus Amplifies Immune Signaling in the CP

**Figure 1:**
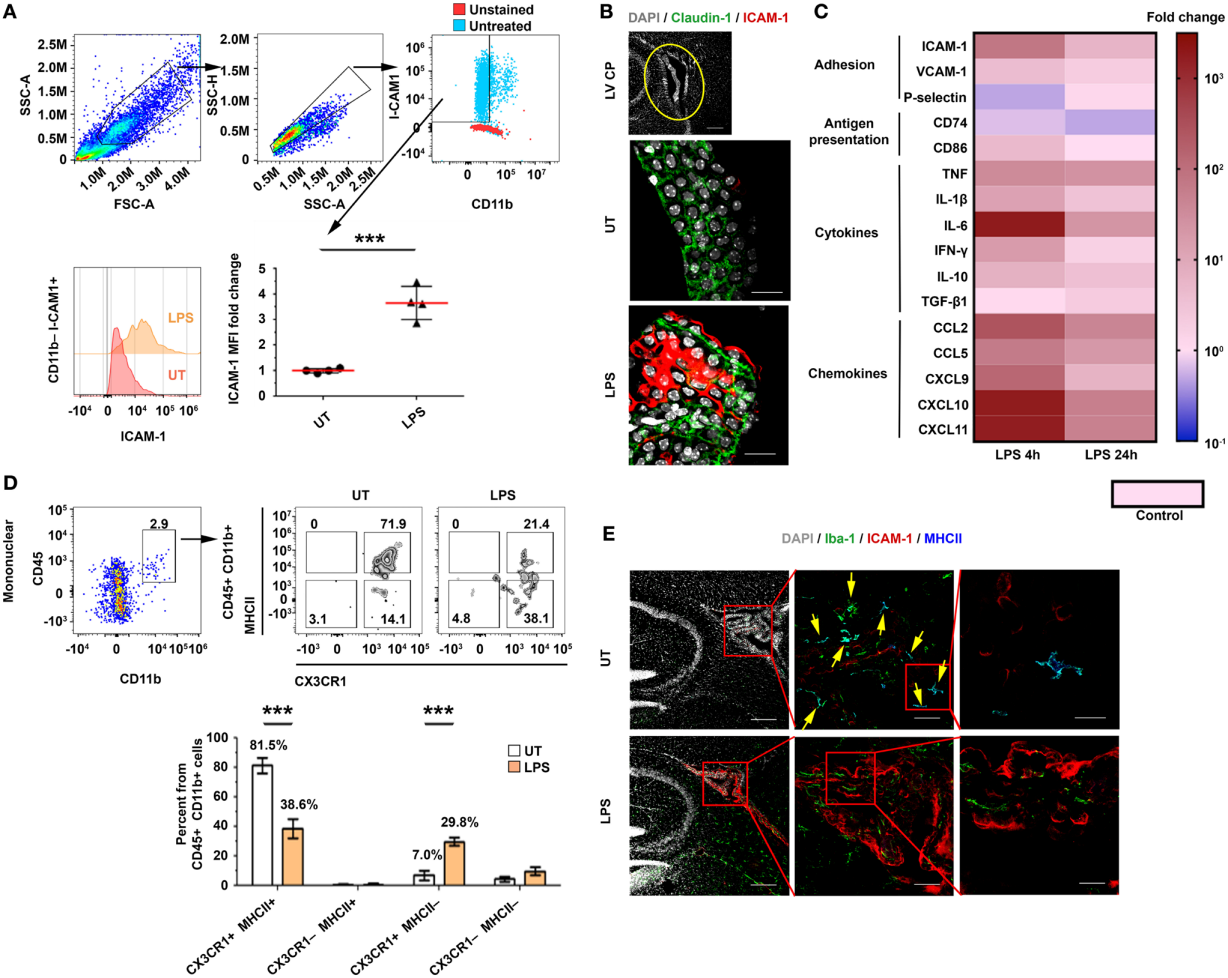
An intraperitoneal (IP) injection of lipopolysaccharide (LPS) activates immune signaling in the choroid plexus (CP). Male C57BL/6 mice were preconditioned with an IP injection of LPS (LPS) or of phosphate-buffered saline (PBS) (Control), or were left untreated (UT). The mice were killed 4 or 24 h later, and their lateral ventricle (LV) CPs were either analyzed by immunohistochemistry (IHC) (24 h) or isolated for quantitative PCR (qPCR) (4 or 24 h) and flow cytometry (24 h) analyses. **(A)** A flow cytometry analysis of the FSC^hi^SSC^hi^CD11b^−^ICAM-1^+^ cell population and the fold change of the median fluorescent intensity (MFI) of intercellular adhesion molecule 1 (ICAM-1) on CP epithelial cells in LPS-preconditioned mice (*n* = 4), as compared with UT mice (*n* = 4). Each symbol represents one LV CP from an individual mouse. **(B)** Representative sagittal brain sections were immunolabeled with anti-Claudin-1 (green) and anti-ICAM-1 (red). DAPI was used for nuclear counterstaining (gray). Sections were then subjected to confocal microscopy and digital image analysis. The z-projection images show a representative LV CP (top panel) and the CP epithelium (green) with an apical expression of ICAM-1 in UT (middle) or LPS-preconditioned (bottom) mice. Scale bars represent 200 µm (top panel) and 20 µm (middle and bottom panels). **(C)** A heat-map representation (fold change from control) of a qPCR analysis of genes encoding key immune mediators in LV CPs, isolated either 4 h (*n* = 5) or 24 h (*n* = 7) after they were preconditioned with either LPS or PBS (Control; *n* = 5). Fold changes and *P* values are provided in Tables S1A,B in Supplementary Material. **(D)** A flow cytometry analysis of the CX_3_CR1 and MHCII subpopulations among the CD45^+^CD11b^+^ mononuclear cells in LPS-preconditioned mice (*n* = 4) and UT wild-type mice (*n* = 4). Bars represent means ± SEM. **(E)** Representative IHC images of LV CPs obtained from the brain sections of UT mice (top), and LPS-preconditioned mice (bottom) immunolabeled with anti-Iba-1 (green), anti-ICAM-1 (red), and anti-MHC II (blue). DAPI was used for nuclear counterstaining (gray). Framed areas are enlarged from left to right. Yellow arrows indicate co-localization of Iba-1 and MHC II. Scale bars represent 200 µm (left), 50 µm (middle), and 10 µm (right). ***P < 0.001 [**(A)** unpaired *t* test; **(D)** two-way ANOVA].

We first identified the extent and kinetics of immune responses in the CP. To this end, we preconditioned mice with an intraperitoneal (IP) injection of a lipopolysaccharide (LPS), perfused them at different time points following the injection, and isolated their lateral ventricle (LV) CPs. A flow cytometry analysis of the CP epithelial cells showed that the levels of ICAM-1 on CP epithelial cells [as measured by median fluorescent intensity (MFI)] significantly increased 24 h after the IP LPS injection (Figure 1A; Figure S1A in Supplementary Material). An immunohistochemistry (IHC) analysis revealed that ICAM-1 is upregulated in Claudin-1^+^ CP epithelial cells, primarily in the apical, CSF-facing surface of the cells, 24 h following the IP LPS injection (Figure 1B). A quantitative PCR (qPCR) analysis revealed a rapid and sharp upregulation of mRNAs that encode immune mediators (Figure 1C) that facilitate leukocyte homing and activation, most notably, of ICAM-1, CD86, the pro-inflammatory cytokines TNF and IFN-γ, and the pro-inflammatory chemokines CCL2, CCL5, and CXCL9–11. The mRNA of most of these genes peaked as early as 4 h following the IP LPS injection (Figure 1C; Tables S1A,B in Supplementary Material).

Next, we determined how immune signaling in the CP impacts its APC subsets. Flow cytometry analyses of mononuclear cells in the CP (Figures S1B–E in Supplementary Material) revealed that, while the IP LPS preconditioning injection did not affect the frequency of CD45^+^CD11b^+^ cells after 24 h (Figure S1F in Supplementary Material), it shifted the population of the CP-resident myeloid cells from 81.5 ± 10.4% CX_3_CR1^+^MHCII^+^ cells to 38.6 ± 13.2% CX_3_CR1^+^MHCII^+^ cells and 29.8 ± 5.7% CX_3_CR1^+^MHCII^−^ cells (Figure 1D). In line with these results, an IHC analysis showed that whereas most Iba-1^+^ myeloid cells in the CP of untreated mice were MHCII^+^ cells (Figure 1E, top panels), the LPS preconditioning reduced the fraction of this subset of cells (Figure 1E, bottom panels). Notably, a marked ICAM-1 upregulation occurred primarily in the epithelial subset rather than the myeloid (Figure 1E) or the endothelial (Figure S2 in Supplementary Material) CP subsets. Overall, these analyses show that, while the peripheral LPS preconditioning injection reduced the myeloid subsets of MHCII-expressing cells in the CP, it markedly increased chemokine and adhesion signals, which facilitate leukocyte homing.

### Activated CD4 T Cells, Injected Intracerebroventricularly (ICV), Home to the CP, and Affect Its Immune Signaling and Leukocytes Homing

**Figure 2:**
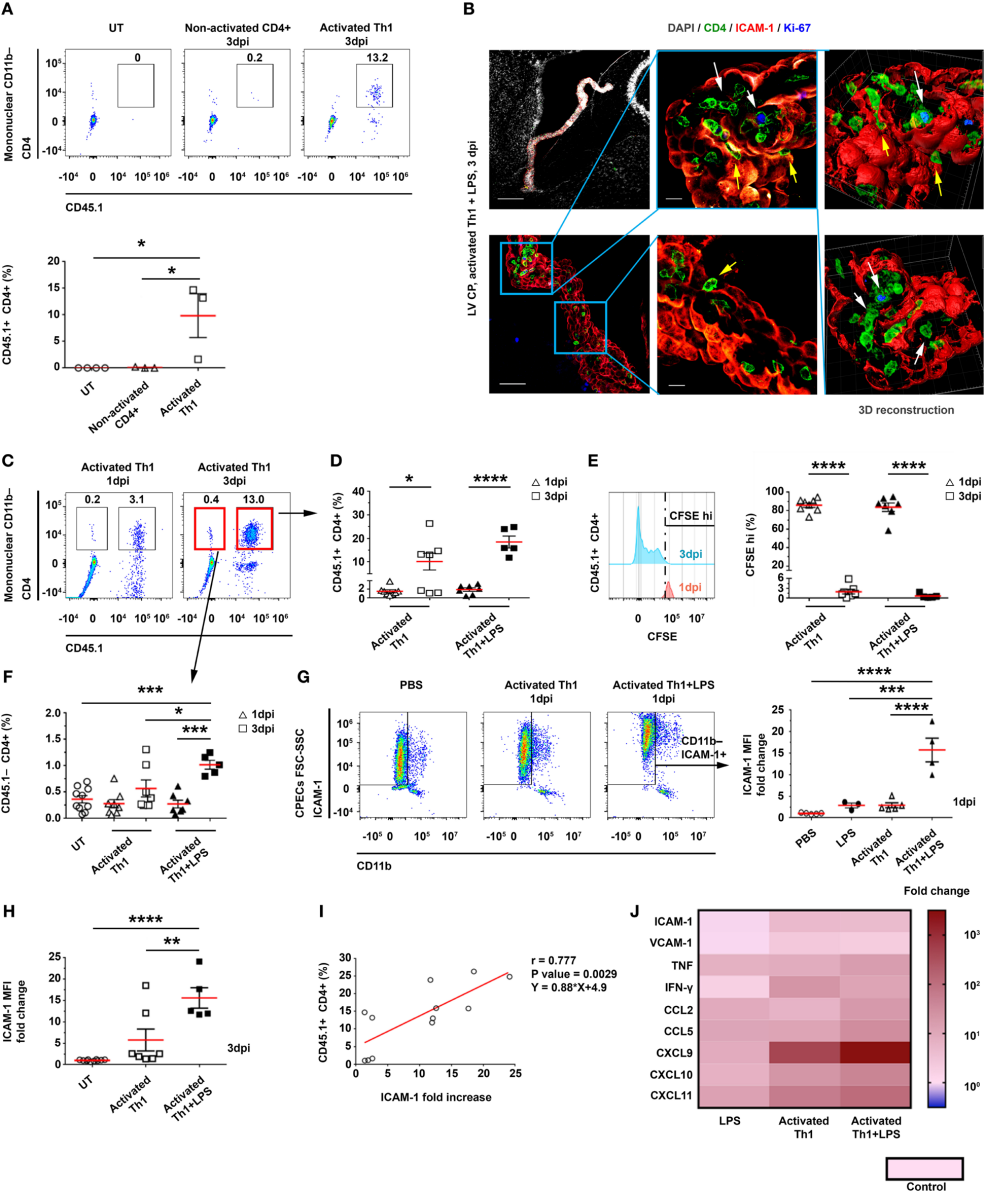
Intracerebroventricularly (ICV) injected activated CD4 T cells adhere to and enter the choroid plexus (CP). CD4 T cells were enriched from CD45.1^+^ splenocytes and ICV-injected, either non-activated (Non-activated CD4^+^) or following activation in a Th1 polarizing cocktail (Activated Th1), to CD45.2^+^ mice that were either untreated (UT) or preconditioned for 24 h with an intraperitoneal injection of lipopolysaccharide (LPS) (+LPS). The lateral ventricle (LV) CPs of these mice were analyzed by flow cytometry 1 and 3 days post-injection (dpi) **(A,C–I)**, by immunohistochemistry (IHC) 3 dpi **(B)**, and by quantitative PCR (qPCR) 1 dpi **(J)**. **(A)** A flow cytometry analysis of gated CD11b^−^ mononuclear cells, showing the frequencies of CD45.1^+^CD4^+^ T cells in UT mice (*n* = 4) and in mice that had been ICV-injected with non-activated CD4 T cells (*n* = 3) or with activated Th1 cells (*n* = 3). **(B)** Representative IHC images, showing the LV CPs in brain sections of LPS-preconditioned mice, 3 dpi of activated Th1 cells immunolabeled with anti-CD4 (green), anti-intercellular adhesion molecule 1 (ICAM-1) (red), anti-Ki-67 (blue), and a DAPI nucleus counterstain (gray). Yellow and white arrows indicate the adherence of CD4 T cells to and their proliferation foci within the CP, respectively, and between ICAM-1^+^ epithelial cell borders. Right panels show 3D reconstructions of z-sections (28 µm overall, 0.7 µm/slice) of the framed area. Scale bars represent 200 µm (top left), 50 µm (bottom left), or 10 µm (middle). **(C–F)** Flow cytometry analyses of LV CPs isolated from mice that had been injected with activated Th1 cells, either without LPS preconditioning (1 dpi, *n* = 8; 3 dpi, *n* = 7), with LPS preconditioning (1 dpi, *n* = 7; 3 dpi, *n* = 5), and UT mice (*n* = 10). **(C)** Flow cytometry plots of gated CD11b^−^ mononuclear cells. **(D)** CD45.1^+^CD4^+^ T-cell frequencies in LV CPs, 1 and 3 dpi. **(E)** CFSE^hi^ frequencies of CD45.1^+^CD4^+^ T cells in LV CPs, 1 and 3 dpi. **(F)** CD45.1^−^CD4^+^ T-cell frequencies in LV CPs, 1 and 3 dpi. **(G)** A flow cytometry analysis of FSC^hi^SSC^hi^CD11b^−^ICAM-1^+^ cells shows the fold change of ICAM-1 median fluorescent intensity (MFI) on CP epithelial cells in mice that had been ICV-injected with activated Th1 cells, either without (1 dpi, *n* = 5) or with LPS preconditioning (1 dpi, *n* = 4), and in mice preconditioned with LPS alone (2 dpi, *n* = 3), compared with mice that had been injected ICV with phosphate-buffered saline (PBS) (1 dpi, *n* = 5). **(H)** Fold change of ICAM-1 MFI in CP epithelial cells in mice with (*n* = 5) or without (*n* = 7) LPS preconditioning and an ICV injection of activated Th1 cells (3 dpi), as compared with UT mice (*n* = 10). **(I)** Correlation between ICAM-1 MFI fold change on CP epithelial cells (compared with untreated mice) and CD45.1^+^CD4^+^ T cells homing to the CPs of UT or LPS-preconditioned mice at 3 dpi (*n* = 12). **(J)** A heat-map representation (fold change from control) of a qPCR analysis of genes encoding immune mediators in LV CPs, which were isolated from mice that had been ICV-injected with PBS (1 dpi, *n* = 5), mice that had been preconditioned with LPS (2 dpi, *n* = 3), or mice that had been injected ICV with activated Th1 cells without (1 dpi, *n* = 5) or with LPS preconditioning (1 dpi, *n* = 4). Fold changes and *P* values are provided in Tables S2A,B in Supplementary Material. Each symbol represents one LV CP from an individual mouse. Bars represent means ± SEM. *P < 0.05, **P < 0.01, ***P < 0.001, and ****P < 0.0001 [**(A,F–H)** one-way ANOVA; **(D,E)** two-way ANOVA; **(I)** Pearson correlation test].

Previous studies in humans have shown that CD4 T cells in the CSF exhibit primarily memory phenotypes and Th1-type chemokine receptors (18–20, 27). To test whether CSF CD4 T cells can home to the apical side of the CP, we ICV injected spleen-derived CD4^+^CD45.1^+^ T cells, which primarily exhibit a memory phenotype, into the LVs of adult CD45.2 C57BL/6 mice. We injected these T cells either non-activated or activated, i.e., 24 h following a polyclonal anti-CD3/anti-CD28 stimulation in the presence of a Th1 polarization cocktail (Figure S3A in Supplementary Material). Then, 3 days post-injection (dpi) of the T cells, we excised the LV CPs of the mice and analyzed them by flow cytometry (Figures S3A,B in Supplementary Material). This analysis revealed that a considerable number of activated Th1 CD4 T cells—but not non-activated CD4 T cells—homed to the CP (Figure 2A). Due to the enhanced immune signaling in the CP following LPS preconditioning (as shown above; Figure 1), we preconditioned the mice with an IP injection of either LPS or phosphate-buffered saline (PBS) as a control (Figure S3A in Supplementary Material), and then injected the mice ICV with activated Th1 cells, labeled with carboxyfluorescein succinimidyl ester (CFSE). Then, either 1 or 3 dpi, we extracted the LV CPs of these mice for further analysis (Figures S3A,B in Supplementary Material). We used IHC to assess, at 3 dpi, the adherence of CD4 T cells to ICAM-1 on the apical surface of the CP epithelial cells, their entry into the CP, and their proliferation inside the CP stroma. Representative images of the LV CPs 3 dpi indicated that the CD4 T cells interact with ICAM-1–expressing CP epithelial cells from the apical side of the epithelial cells (yellow arrows in Figure 2B). We also observed foci of proliferating Ki-67^+^CD4 T cells within the CP stroma (indicated by white arrows in Figure 2B). Flow cytometry analyses revealed that most exogenous CD45.1^+^CD4 T cells that homed to the CP at 1 dpi were CFSE^hi^ non-proliferating cells, while the proportion of CD45.1^+^CD4^+^CFSE^neg/low^ proliferating T cells among the CD11b^−^ mononuclear cells increased significantly at 3 dpi (Figures 2C–E). Although the LPS preconditioning did not affect proliferation, it increased the frequency of mice showing a substantial homing of the T cells to the CP at 3 dpi (Figures 2C–E). Notably, at 3 dpi, the CPs of LPS-preconditioned mice showed more endogenous CD45.1^−^CD4 T cells than the CPs of untreated mice and of LPS-preconditioned mice at 1 dpi; this finding indicates that CSF-activated T cells not only home to the CP but also induce a milieu that trigger the entry of endogenous circulating T cells into it (Figures 2C,F).

At 3 dpi, ICAM-1 was expressed significantly more in the CP than in the blood–leptomeningeal barrier (BLMB) and the blood–brain barrier (BBB) (Figure 2B; Figure S3C in Supplementary Material). A flow cytometry analysis of ICAM-1 expression in CP epithelial cells at 1 dpi showed that the IP LPS preconditioning and the ICV injection of activated Th1 T cells had a marked synergistic effect (as compared with untreated mice, the MFI was 15.7 ± 5.5-fold higher for mice preconditioned with LPS and ICV-injected with activated Th1 cells, as compared with a 2.9 ± 1.3-fold increase in mice preconditioned with LPS and not injected with Th1 cells, and with a 2.9 ± 1.0-fold increase in mice ICV-injected with activated Th1 cells but not preconditioned with LPS; Figure 2G). This synergistic effect was still observed at 3 dpi (Figure 2H). A significant positive Pearson correlation was found between the frequency of CD45.1^+^ T cells (Figure 2D) and the normalized MFI of ICAM-1 in CP epithelial cells (Figure 2H) at 3 dpi (*r* = 0.777, *P* = 0.0029; Figure 2I). A qPCR analysis of mRNAs isolated from LV CPs of mice 1 dpi revealed that the LPS preconditioning combined with the ICV injection of Th1 cells upregulated inflammatory signals in the CP, which supports leukocyte homing (Figure 2J; Tables S2A,B in Supplementary Material).

### The Entrance of CD4 T Cells to the CP Enhance Antigen Presentation

**Figure 3:**
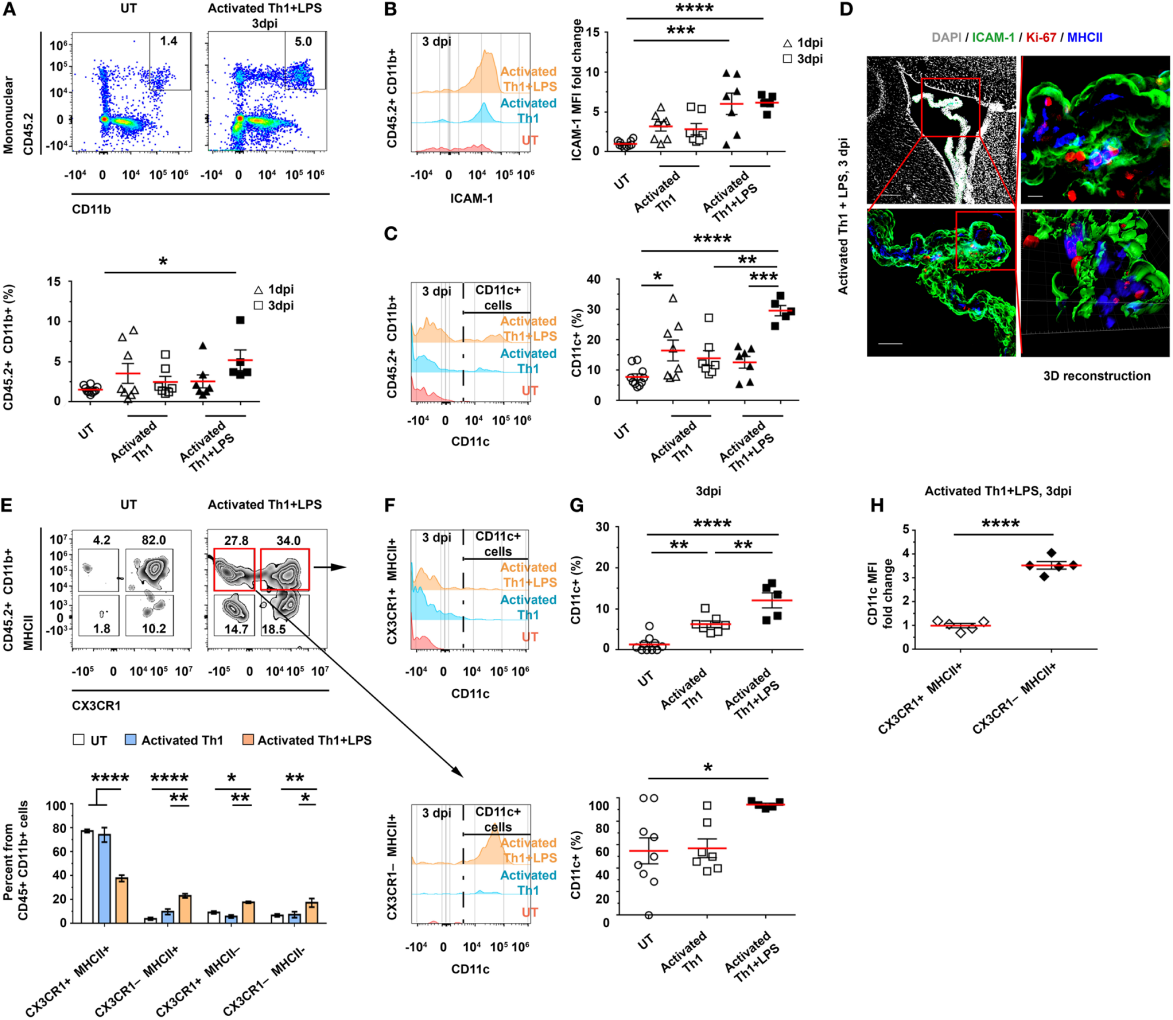
CD4 T cells homing to the choroid plexus (CP) locally impact antigen-presenting cell subsets. Activated CD45.1^+^ Th1 cells were intracerebroventricularly (ICV)-injected into the lateral ventricles (LVs) of either untreated (UT) or lipopolysaccharide (LPS)-preconditioned (+LPS) CD45.2 wild-type mice. The LV CPs of these mice were then analyzed with flow cytometry, either 1 days post-injection (dpi) **(A–C)** or 3 dpi **(A–C,E–H)**, and with immunohistochemistry (IHC) 3 dpi **(D)**. Flow cytometry analyses of gated CD45.2^+^CD11b^+^ mononuclear myeloid cells of LV CPs isolated from UT mice (*n* = 10) or from mice that had been ICV-injected with Th1 cells, either with (1 dpi, *n* = 7; 3 dpi, *n* = 5) or without (1 dpi, *n* = 8; 3 dpi, *n* = 7) LPS preconditioning. **(A,B)** The frequency of CD45.2^+^CD11b^+^ mononuclear myeloid cells in individual mice **(A)** and their expression levels of intercellular adhesion molecule 1 (ICAM-1) **(B)**. **(C)** The frequency of CD11c subsets among the myeloid population. **(D)** Representative IHC images, showing the LV CPs of mice that were preconditioned with LPS and ICV-injected with Th1 cells. Images were taken 3 dpi and immunolabeled with anti-ICAM-1 (green), anti-Ki-67 (red), anti-MHC II (blue), and a DAPI nuclear counterstain (gray). Scale bars: 200 µm (top left), 50 µm (bottom left), and 10 µm (top right). Bottom right panel shows a 3D reconstruction of z-sections (27.3 µm overall, 0.7 µm/slice), of the framed area. **(E)** The distribution of CX_3_CR1 and MHCII cells among the CD45.2^+^CD11b^+^ myeloid subsets of the CP at 3 dpi. **(F,G)** The frequency of CD11c^+^ cells among CX_3_CR1^+^MHCII^+^ cells (top panels) and CX_3_CR1^−^MHCII^+^ cells (bottom panels). **(H)** CD11c median fluorescent intensity (MFI) in CX_3_CR1^+^MHCII^+^ and CX_3_CR1^−^MHCII^+^ in mice that had been LPS preconditioned and ICV injected with activated Th1 cells. Each symbol represents one LV CP from an individual mouse. Bars represent means ± SEM. *P < 0.05, **P < 0.01, and ***P < 0.001, ****P < 0.0001 [**(A–C,G)** one-way ANOVA; **(E)** two-way ANOVA; **(H)** unpaired *t* test].

The homing of CD4 T cells to—and their transmigration into—the CP suggests that they can undergo antigen-specific stimulation by either CP-resident or newly arriving APCs. Thus, we sought to determine how the initial homing of the Th1 cells to the CP locally shapes the profile of APCs. To this end, we ICV-injected activated CD45.1^+^ Th1 cells to C57BL/6 mice, with or without LPS preconditioning (injected IP), as described earlier (Figure S3A in Supplementary Material). Then, at either 1 or 3 dpi, we isolated the LV CPs of these mice and analyzed their APCs by flow cytometry. As compared with untreated mice, the LPS-preconditioned, Th1 cells-injected mice showed no change in the total number of CD45.2^+^CD11b^+^ cells at 1 dpi; however, at 3 dpi, they showed a 3.4 ± 1.9-fold increase in the frequency of these cells (Figure 3A), and the levels of CD45.2, CD11b (Figures S4A,B in Supplementary Material), and ICAM-1 (Figure 3B) in these cells were upregulated. Notably, as compared with untreated mice, mice injected with activated Th1 cells showed, at 1 dpi, a significantly higher frequency of CD11c^+^ cells among the CD45.2^+^CD11b^+^ cells, and this frequency was even further increased at 3 dpi specifically when the mice were preconditioned with LPS before the injection of the activated Th1 cells (Figure 3C). An IHC analysis of the LV CP showed a high prevalence of Iba-1^+^ cells exhibiting the MHCII^+^ phenotype, with a higher expression of CD11c, in LPS-preconditioned, Th1 cells-injected mice, as compared with untreated mice (Figure S4C in Supplementary Material). Most MHCII^+^ cells in the treated mice were Ki-67^−^, suggesting that they were newly recruited following the ICV injection of the Th1 cells (Figure 3D).

The change in CP-resident myeloid cells can greatly alter the antigen-presentation capacity of the CP-resident cells. Under normal conditions, about 80% of the CP-resident CD45^+^CD11b^+^ cells are CX_3_CR1^+^MHCII^+^ (Figures S1B–E in Supplementary Material). However, 3 days after the injection of activated Th1 cells to LPS-preconditioned mice, we found a marked decrease in the frequency of the CX_3_CR1^+^MHCII^+^ population and a concomitant increase in the frequency of the CX_3_CR1^−^MHCII^−^, CX3CR1^−^MHCII^+^, and CX_3_CR1^+^MHCII^−^ populations (Figure 3E). Among the MHCII^+^ cells, the frequency of CD11c^+^ cells was increased at 3 dpi (Figures 3F,G) and reached 12.1 ± 4.1% among the CX_3_CR1^+^MHCII^+^ cells (compared with 1.3 ± 1.8% in untreated mice) and 94.4 ± 2.8% among the CX_3_CR1^−^MHCII^+^ cells (compared with 55.0 ± 33.1% in untreated mice). Notably, the frequency of CX_3_CR1^−^MHCII^+^ cells among all CD45.2^+^CD11b^+^ mononuclear cells, which were almost absent in untreated mice (4.0 ± 3.3%), significantly increased at 3 dpi (namely, to 23.2 ± 3.7%) in LPS-preconditioned, Th1 cells-injected mice (Figure 3E), with increased levels of CD11c (a 3.4 ± 0.4-fold increase in CX_3_CR1^−^MHCII^+^, as compared with CX_3_CR1^+^MHCII^+^ cells; Figure 3H). Taken together, these findings suggest that the homing of activated Th1 cells to the CP locally facilitates antigen presentation, manifested by an increased frequency of CD11c^+^MHCII^+^ cells (expressing higher levels of ICAM-1 and CD11c) among the CP myeloid subsets.

### Resting CSF T Cells Undergo Antigen-Specific Activation Within the CP

The findings presented above indicate that ICV-injected activated Th1 cells home to the CP and transmigrate into its stroma, where they proliferate and locally alter the immune milieu. However, it was unclear whether resting T cells in the CSF could be recruited to the CP and be stimulated by an antigen present in the CSF. To address this issue, and in line with previous studies (9, 15, 21) and our own data, which show the impact of IFN-γ and Th1 signaling on antigen presentation in the CP, we intrathecally co-injected IFN-γ with either ovalbumin (OVA), myelin oligodendrocyte glycoprotein (MOG), or PBS (Control) intra-cisterna magna (ICM) of C57BL/6 mice. Then, after 24 h, we ICV-injected the mice with CFSE-labeled resting OVA-specific Th1 cells, together with OVA, MOG, or PBS. Finally, 3 days later, we perfused the mice with PBS and harvested their brains, CPs, and spleens (Figure S5A in Supplementary Material).

**Figure 4:**
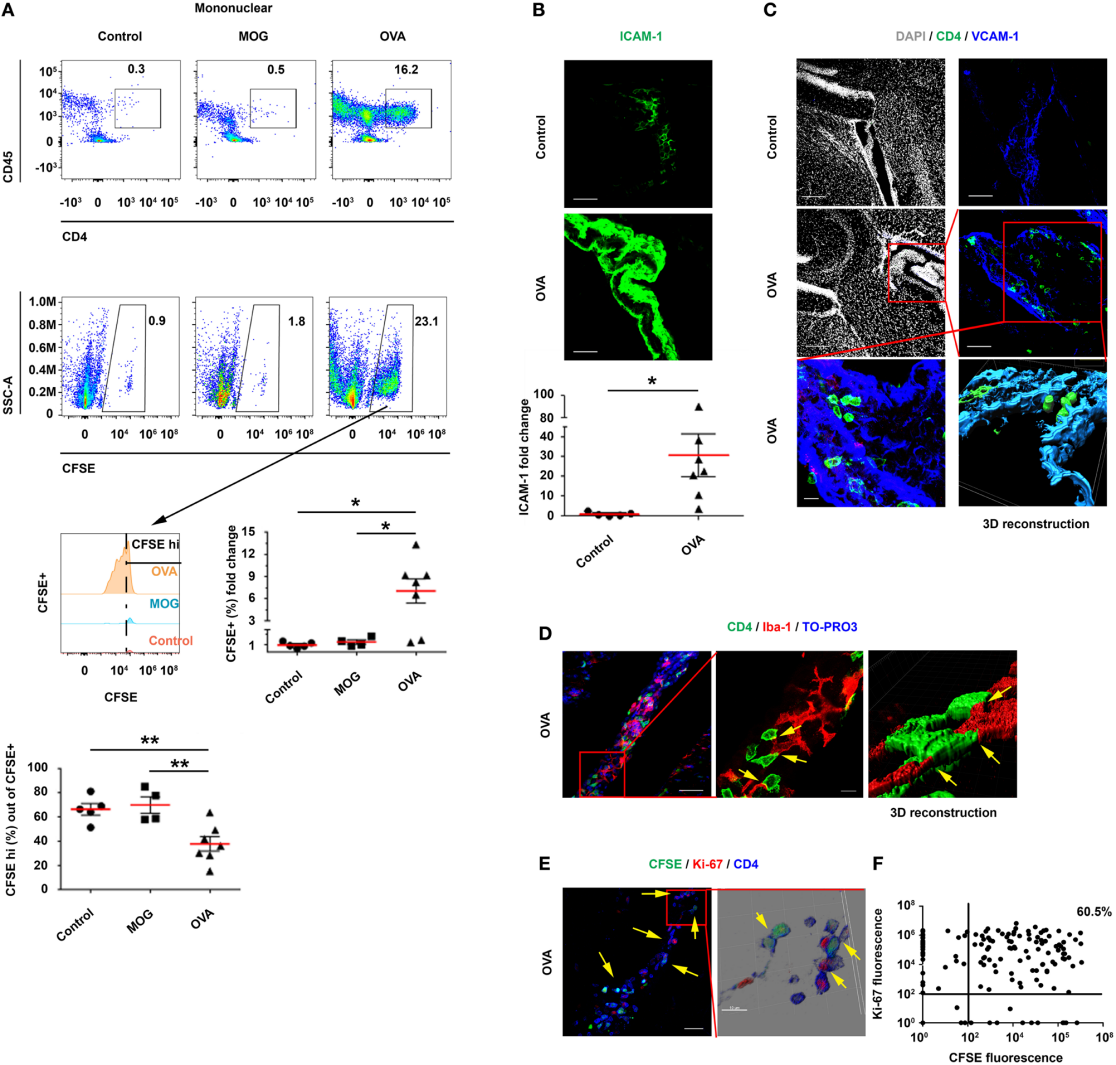
Intracerebroventricularly (ICV)-injected, resting ovalbumin (OVA)-specific CD4 T cells undergo proliferation within the choroid plexus (CP) in a cerebrospinal fluid-antigen-dependent manner. Interferon gamma was injected intra-cisterna magna to wild-type mice, either alone or together with OVA_323–339_ or MOG_35–55_ (as a control peptide). After 24 h, the mice were ICV-injected with carboxyfluorescein succinimidyl ester (CFSE)-labeled, resting OVA-specific Th1 cells, either without a peptide (Control; *n* = 5), with OVA_323–339_ (OVA; *n* = 7), or with MOG_35–55_ [myelin oligodendrocyte glycoprotein (MOG); *n* = 4]. At 3-day post-injection, the lateral ventricle (LV) CPs of these mice were analyzed by flow cytometry and immunohistochemistry (IHC). **(A)** Flow cytometry of isolated CPs. The cellular fraction gated on mononuclear cells shows CD45^+^CD4^+^ and CFSE^+^ T cells. **(B–F)** IHC images of LV CPs obtained from the control and from the OVA-injected mice. **(B)** Representative brain sections of OVA-injected and control mice, immunolabeled with anti-intercellular adhesion molecule 1 (ICAM-1) (green). The graph shows fold change in ICAM-1 expression in the LV CPs of OVA-injected mice, normalized to the control mice. Scale bars represent 50 µm. **(C)** Representative brain sections of OVA-injected and control mice, immunolabeled with anti-CD4 (green), anti-vascular cell adhesion molecule 1 (VCAM-1) (blue), and a DAPI counterstained (gray). Scale bars represent 200 µm (top left and middle left panels), 50 µm (top right and middle right panels), and 10 µm (bottom left panel). The bottom right panel shows a 3D reconstruction of z-sections (25.9 µm overall, 0.7 µm/slice) of the framed area. **(D–F)** Analyses of the interactions between T cells and myeloid cells, and the proliferation of T cells within the CP. **(D)** Representative brain sections of OVA-injected mice, immunolabeled with anti-CD4 (green), anti-Iba-1 (red), and a TO-PRO-3 nuclear counterstain (blue). The yellow arrows indicate co-localization of CD4 and Iba-1. The right panel shows a 3D reconstruction of z-sections (9.5 µm overall, 0.5 µm/slice) of the framed area. Scale bars represent 50 µm (left) and 10 µm (middle). **(E)** Representative brain sections of OVA-injected mice immunolabeled with anti-Ki-67 (red) and anti-CD4 (blue). The yellow arrows indicate proliferating CD4 T cells in the CP. The right panel shows a 3D reconstruction of z-sections (22 µm overall, 2 µm/slice) of the framed area. Scale bars represent 50 µm (left) and 10 µm (right). **(F)** Expression pattern of CFSE and Ki-67 in CD4^+^ T cells, which were detected in three 40-µm brain sections of a single LV CP from a mouse injected with OVA and with OVA-specific T cells. **(A,B)** Each symbol represents one LV CP from an individual mouse. Bars represent means ± SEM. *P < 0.05, **P < 0.01 [**(A)** one-way ANOVA, **(B)** unpaired *t* test].

A flow cytometry analysis revealed a marked homing of CD45^+^CD4^+^ T cells to the LV CPs of OVA-injected mice, with a significantly higher frequency of CFSE^+^ T cells in these mice, as compared with MOG-injected mice and control mice (Figure 4A). In addition, the gating of CFSE^+^ T cells showed that these cells underwent proliferation only in the OVA-injected mice, as indicated by a significant decrease in the proportion of CFSE^hi^ cells and by the number of proliferation cycles in the CFSE histogram (Figure 4A). IHC images of the LV CP revealed a 30.7-fold increase in the expression of ICAM-1 on CP epithelial cells in the OVA-injected mice, as compared with the control mice (Figure 4B). CD4 T cells were observed inside the CP stroma (in between the borders of the epithelial VCAM-1^+^ cells) in the OVA-injected mice but not in the control mice (Figure 4C). Images of representative brain sections and a 3D reconstruction showed the CD4 T cells interacting with CP myeloid (Iba-1^+^) cells in the CP stroma, presumably generating immunological synapses (see yellow arrows in Figure 4D; Figure S5D in Supplementary Material). The CD4 T cells in the CPs of OVA-injected mice showed a differential expression of Ki-67 and CFSE, indicating various stages of proliferation (yellow arrows in Figures 4E,F; Figure S5B in Supplementary Material). Notably, whereas we could not find the ICV-injected T cells in the spleen (Figure S5C in Supplementary Material) or in parenchymal blood vessels (Figure S5E in Supplementary Material), we detected them crossing the ependymal layer of the ventricles into the parenchyma (Figure S5F in Supplementary Material) and found some cells in the meninges and in the cortex parenchyma (Figure S5G in Supplementary Material). These findings are in line with the shift of CP APCs toward an increased frequency of DC-like CD11c^+^MHCII^+^ myeloid cells (Figure S6 in Supplementary Material), as we also observed following the ICV injection of activated Th1 cells (see Figure 3).

### The Homing of Activated Th1 Cells to the Apical Surface of the CP Is Chemokine and ICAM-1 Dependent

Which signals mediate the initial adherence and the homing of CD4 T cells to the apical surface of the CP? As reported above, we found that a peripheral LPS stimulation evokes an immediate and tightly controlled upregulation of chemokines and of ICAM-1 and VCAM-1 at the apical surface of the CP epithelium; hence, we sought to address the roles of these molecules by using a novel *ex vivo* culture system. To this end, we isolated intact CPs from untreated C57BL/6 mice (without prior perfusion, to preserve the microstructures of the CP) and cocultured them with activated T cells in artificial CSF (aCSF) (Figure S7A in Supplementary Material). To begin exploring the recruitment process, we cultured the CPs either with CFSE-labeled, non-activated CD4 T cells or with activated Th1 cells from CD45.1 mice (Figure S7A in Supplementary Material). After 24 h, we collected the CPs, washed them vigorously, and subjected them to flow cytometry.

**Figure 5:**
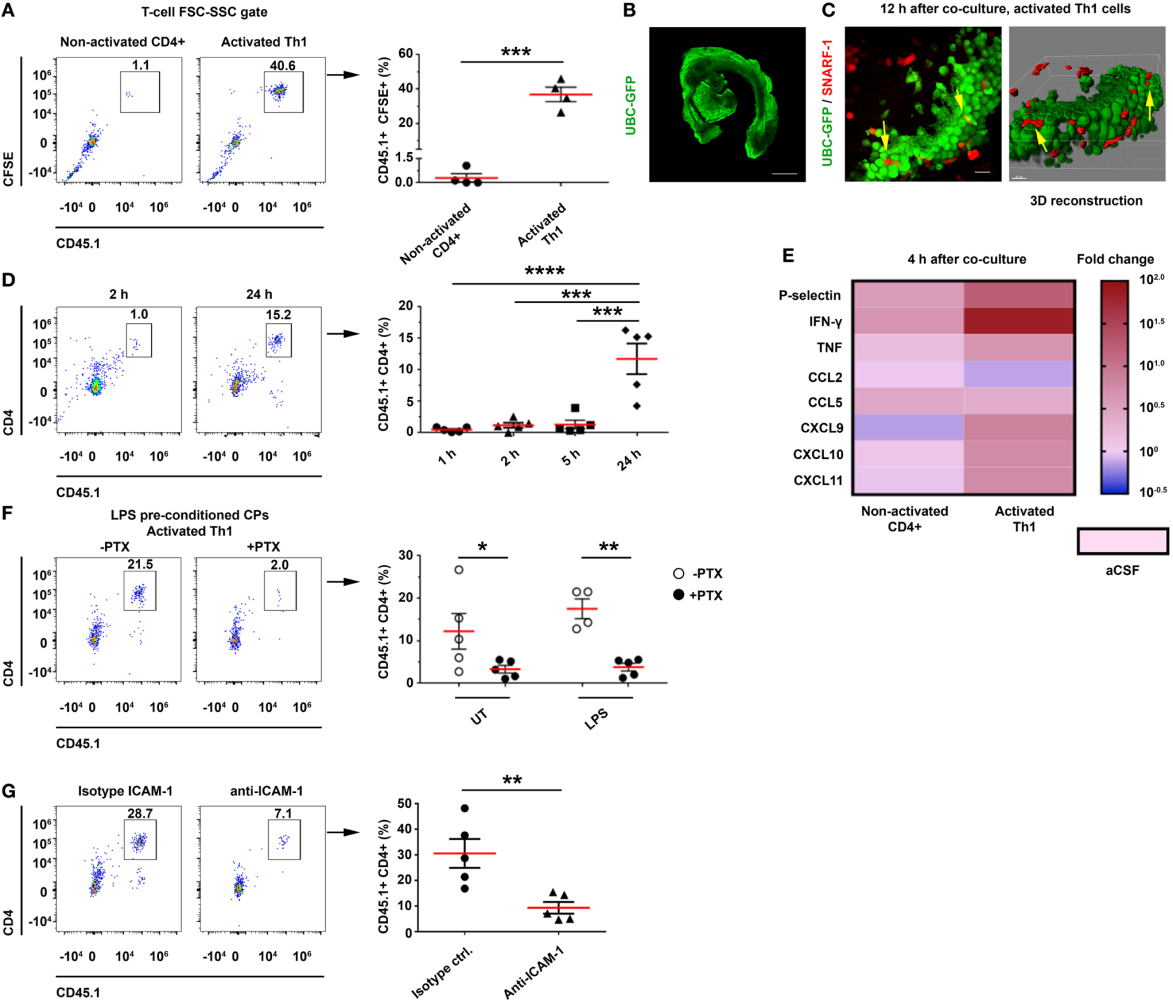
CD4 T cells home to the choroid plexus (CP) in an activation-, chemokine-, and intercellular adhesion molecule 1 (ICAM-1)-dependant manner. Intact lateral ventricle (LV) CPs from non-perfused CD45.2 mice, with or without lipopolysaccharide (LPS) preconditioning, were cocultured *ex vivo* with CD45.1 CD4 T cells in artificial CSF (aCSF) and then analyzed with flow cytometry and confocal microscopy. **(A–E)** *Ex vivo* cocultures, showing the homing of activated T cells to the CP with flow cytometry and live-cell imaging, and their impact on gene expression. **(A)** Flow cytometry analysis of the FSC-SSC T-cell population in untreated (UT) CPs cocultured for 24 h with CD45.1^+^ carboxyfluorescein succinimidyl ester-labeled non-activated (Non-activated CD4^+^; *n* = 4) or activated (Activated Th1; *n* = 4) T cells. **(B,C)** Confocal live-cell imaging of UT, GFP-labeled CPs, which were derived from UBC-GFP mice **(B)** and cocultured with SNARF-1^+^ Th1 cells adhering to and migrating within the CP [**(C)**; yellow arrows indicate interactions]. Scale bars represent 500 µm **(B)**, 20 µm **(C)**. **(D)** Homing kinetics of activated Th1 cells to the CP, after 1, 2, 5, and 24 h (*n* = 5 at each time point) of *ex vivo* coculturing with untreated CPs. **(E)** A heat-map representation (fold change from control aCSF) of a quantitative PCR analysis of genes encoding immune mediators, performed on total RNA isolated from LV CPs that were cocultured with either non-activated CD4^+^ (*n* = 4) or activated (*n* = 4) Th1 cells, as compared with an aCSF control (*n* = 3), 4 h after initiating the coculture. Fold changes and *P* values are provided in Tables S3A,B in Supplementary Material. **(F,G)** The role of chemokines and cell adhesion molecules in T-cell homing to the CP. **(F)** LV CPs were isolated from mice with or without LPS preconditioning, and they were then cocultured for 24 h with CD45.1 activated Th1 cells, either with a prior pre-treatment of the chemokine-signaling inhibitor pertussis toxin (PTX) (*n* = 5 for LPS-preconditioned and *n* = 5 for phosphate-buffered saline-injected mice, respectively), or without it (*n* = 5 and *n* = 4, respectively). Then, the CPs were analyzed by flow cytometry. The graph shows the frequency of CP-homing T cells. **(G)** CPs were isolated from LPS-preconditioned mice and then cultured for 24 h with CD45.1 activated Th1 cells in the presence of ICAM-1 neutralizing antibodies, or an isotype control (*n* = 5 in each group). The graph shows the frequency of CP-homing T cells. Each symbol represents one LV CP from an individual mouse. Bars represent means ± SEM. *P < 0.05, **P < 0.01, ***P < 0.001, and ****P < 0.0001 [**(A,G)** unpaired *t* test; **(D)** one-way ANOVA; **(F)** two-way ANOVA].

The flow cytometry analysis revealed a significant homing of CFSE^+^CD45.1^+^ activated Th1 cells—but not of non-activated CD4 T cells—to the CP (Figure 5A; Figure S7B in Supplementary Material). To examine the ability of the Th1 cells to migrate into the CP from its apical side, we labeled activated Th1 cells with SNARF-1 (a red cell tracer) and cocultured them with CPs from UBC-GFP mice (Figure 5B) for live imaging (Figure S7A in Supplementary Material). Confocal microscopy images were taken 12 h later and showed the SNARF-1^+^ activated Th1 cells interacting with the CP epithelial cells (see yellow arrows in Figure 5C). A live-cell imaging showed that CX_3_CR1^+^ cells are widely distributed in the CP (Figure S7C in Supplementary Material), where they interact for more than 2 h with the SNARF-1^+^ activated Th1 cells on the apical surface of the CP (Figure S7D and Video S1 in Supplementary Material), presumably as an initial stage before transmigrating to the CP stroma. The flow cytometry analysis revealed a low level of T-cell homing during the first 5 h following coculturing the Th1 cells with CPs, but a significant homing to the CP at 24 h (Figure 5D). This finding suggests that CP preconditioning (i.e., the increase in CP chemokines) enhances homing. A qPCR analysis revealed that the Th1 chemokines and cytokines that are required for leukocyte homing were upregulated 4 h after coculturing the CPs with the activated—but not with the non-activated—T cells (Figure 5E; Tables S3A,B in Supplementary Material). To evaluate the role of chemokine signaling in the homing of the T cells to the CP, we pretreated activated Th1 cells with pertussis toxin (PTX) before coculturing them with intact CPs, which were isolated from either untreated mice, LPS-preconditioned mice, or PBS-preconditioned mice. A flow cytometry analysis performed 24 h after the initiation of the coculture showed that PTX abolished T-cell homing to the CP, regardless of LPS preconditioning (Figure 5F). Similarly, ICAM-1 blocking antibodies significantly reduced T-cell homing to the CP (Figure 5G), but the addition of anti-VCAM-1 antibodies did not reduce it further (data not shown).

## Discussion

**Figure 6:**
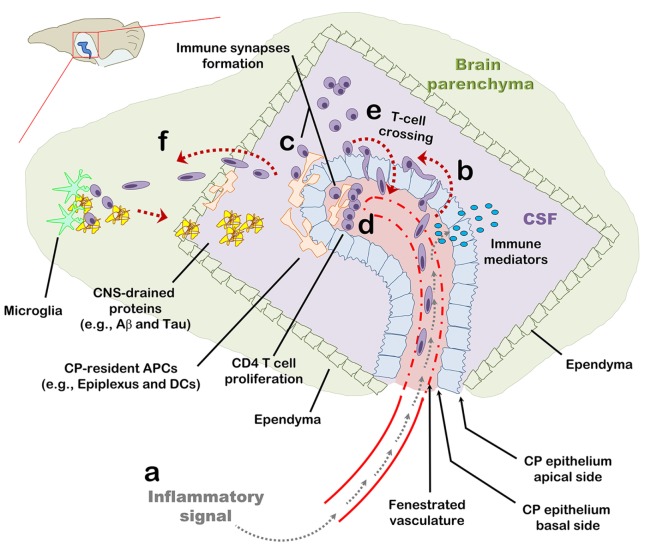
The choroid plexus (CP) as a checkpoint for cell-mediated immunity in the central nervous system (CNS): a suggested model. The CP manufactures most of the cerebrospinal fluid (CSF) and serves as an interface between the blood and the CNS. **(A)** The CP primarily comprises a fenestrated vasculature, a stroma, and epithelial, whose apical surfaces face the CSF. Inflammatory signals such as IL-1β and tumor necrosis factor activate the CP vasculature and epithelium and induce immune signaling in the CP compartment. **(B)** As part of this inflammatory reaction, peripheral blood effector and/or memory T cells are recruited to the CP stroma and into the CSF. **(C,D)** Antigens in the CNS, either self or foreign, which drain into the CSF, are sampled by antigen-presenting cells and presented to CD4 T cells which, thereby, undergo activation and migrate into the CNS parenchyma. **(E)** Intercellular adhesion molecule 1 and chemokines strongly upregulated at the apical surface of the CP epithelium allow T cells in the CSF to adhere the CP and cross its epithelium back into the CP stroma. **(F)** Activated CD4 T cells further facilitate cell-mediated immunity in the CNS by preconditioning the CNS for cell migration across the ependymal layer of the ventricle and/or across the parenchymal and meningeal CNS vasculature. T-cell activation in the CP compartment, may not only serve as a checkpoint for cell-mediated immunity in the CNS but also impact the immune network required for brain functioning and repair at steady-state.

This study demonstrates that, following an IP injection of LPS, within hours, the CP is preconditioned to support homing of CD4 T cells, a phenomenon that at least partially depends on adhesion molecules and chemokine signaling. Provided that their cognate peptide is present, T cells in the CP undergo stimulation and proliferation, which locally shift the immune milieu and the monocyte/DC subsets so as to facilitate leukocyte recruitment and antigen presentation. Our findings suggest that the CP functions as a harbor for T cells, where their antigen-specific activation has both a short- and long-term impact on cell-mediated immunity in the CNS (Figure 6).

The fenestrated capillaries of the CP provide an access point for blood-borne mediators (5, 11, 12). Peripheral immune stimuli activate the CP and upregulate homing molecules such as chemokines and cell adhesion molecules within this compartment (11, 12). Immune signaling in the CP has thus been considered to be a gateway for leukocyte trafficking from the peripheral blood into the CNS (10, 23, 28–33). Our findings suggest an additional route for leukocyte trafficking, namely, from the CSF directly to the CP. We show that activated ICV-injected CD4 T cells adhere to the apical surface of the CP epithelium in a chemokine- and ICAM-1-dependent manner. Following immune activation and the homing of activated Th1 cells to the CP, CCL5 and CXCL9–11 (the ligands of CCR5 and CXCR3, respectively) are upregulated and facilitate the homing process. Such an upregulation of chemokines in the CP, along with the expression of ICAM-1 at the apical surface of the epithelial cells, suggest that as part of being a compartment that propagates peripheral inflammation into the CNS (11, 12, 34), the CP also serves as a harbor for T cells which presumably participate in this process (10, 19).

The marked homing of ICV-injected Th1 cells to the CP prompted us to investigate whether these cells can undergo local stimulation and proliferation. Such a response would suggest that the CP can serve as a niche for presentation of CNS antigens, draining from the interstitial fluid to the CSF (5, 17, 35, 36), to T cells. Our data indeed demonstrate that ICV-injected Th1 cells not only adhere to the CP epithelium, where they can interact with epiplexus macrophages, but they also transmigrate into the CP stroma and form tight interactions with myeloid cells—resulting in T-cell proliferation within the CP. Alongside this process, we found a marked increase in the antigen-presentation capacity of the CP, which was manifested as an upregulation of CD11c by the dominant resident CX_3_CR1^+^MHCII^+^ myeloid cell population in the CP. We also report the appearance of a new CP myeloid subset: CD45^high^CD11b^+^CX_3_CR1^−^MHCII^+^CD11c^high^. These APCs show upregulated levels of ICAM-1, which is a key player in the formation of the immunological synapse facilitating long-lasting, antigen-specific T-cell priming by DCs (37, 38). Recent studies that examined CP macrophages (primarily by using the Cx3cr1^GFP/WT^ reporter mouse) have not observed this myeloid subset (6, 17, 33), which may be essential for facilitating antigen-dependent T-cell stimulation in the CP. The characteristics of this subset of cells, in terms of their recruitment process, function, and turnover in the CP, should be studied further.

What is the physiological context in which T cells home to the CP and regulate CNS inflammation in health and disease? Provided that the CP consists of fenestrated capillaries, it is, perhaps, among the first compartments within the CNS to react to blood-circulating inflammatory signals with a prompt and robust upregulation of pro-inflammatory genes, which can be disseminated through the CSF and impact brain inflammation (11, 34). In addition, blood vessels in the CP constitutively express P- and E-selectin which may allow the extravasation of CD45RO^+^ peripheral blood lymphocytes expressing the P-selectin ligand, PSGL-1 (2, 19). Our results demonstrate that based on antigen availability, such CP-homing T cells can undergo activation at the CP stroma by local and/or infiltrating APCs and significantly impact the immune milieu in a manner which can possibly precondition the CNS for leukocyte trafficking (Figure 6) (10, 15, 21, 39). Furthermore, we show that T-cell activation within the LV results in their migration to the brain parenchyma across the ependymal layer. Given that the brain contains lymphatic vessels that drain the interstitial fluid and CSF into the deep cervical lymph nodes (dCLNs) (40–42). It is thus plausible that naïve T cells undergoing activation in dCLNs with brain-draining antigens undergo restimulation at the CP compartment by their cognate antigens as part of the recruitment process into the brain parenchyma (21, 30). The CP may thus serve as an early immune checkpoint before peripherally activated T cells migrate into the CNS parenchyma. This suggestion is in line with a recent study in a mouse model of stroke showing that T cells migrate to the brain parenchyma primarily from the CP (31). While the mechanism whereby T cells cross the CP into the brain parenchyma requires further research, our results suggest that at least in some inflammatory processes in the CNS, T-cell activation in the CP compartment may take place before leukocytes cross the CP epithelium and subsequently the ependymal layer of the ventricle.

Our results surprisingly show that T cells not only migrate to the CP from peripheral blood but they also adhere to and cross the apical surface of the CP along with IFN-γ signaling and a marked upregulation of ICAM-1 at the CP epithelium. In humans, there are about 1,000–3,000 leukocytes per milliliter CSF, comprising primarily memory/effector CD4 T cells crossing the CP, BBB, or BLMB under both health and disease (17–20, 26). We suggest that during brain inflammation CD4 T cells, which cross the CP epithelium, can harbor the CP and undergo restimulation as a way to facilitate and/or modulate the inflammatory process in the CNS (Figure 6). Furthermore, it is possible that such interactions of CSF T cells with the CP underlie various aspects of immune mechanisms in the CNS such as through the course of multiple sclerosis, Alzheimer’s disease, and stroke (26, 31, 43). One example is IFN-γ signaling (resulting primarily from activation of Th1 cells), which was recently found to be crucial for maintaining beneficial immunity and proper brain functioning (e.g., clearance of misfolded proteins, modulation of neurotoxic inflammation, release of neurotrophic factors, and social performance) during aging and the progression of Alzheimer’s disease (9, 15, 21, 28, 29, 44). Future studies targeting immune signaling specifically in various cell subsets of the CP will allow a better understanding of mechanisms by which the CP may impact various disease and infectious processes in the CNS.

Taken together, our data indicate that the CP is a CNS compartment that rapidly responds to peripheral inflammation and thereby triggers immune signaling. The constitutive presence of homing molecules, APCs, and CNS antigens generates a niche where T cells undergo proliferation and stimulation, and, thereby, may direct and amplify immune pathways in the CNS. Such cellular and molecular events in the CP may provide new avenues for intervening in neurological disorders.

## Materials and Methods

### Mice

Adult male C57BL/6 wild-type mice, CD45.1 mice (stock number 002014), OT-II TCR Tg mice (stock number 004194), and UBC-GFP mice (stock number 004353) were purchased from the Jackson Laboratory (Bar Harbor, ME, USA). Cx3cr1^GFP/WT^ mice were kindly donated by Prof. Steffen Jung (Weizmann Institute of Science, Israel). The number of mice in each experimental group varies according to the standard deviation obtained in each of the experiment. All surgical and experimental procedures were approved by the Institutional Animal Care and Use Committee of Ben-Gurion University of the Negev, Israel (Approval Number IL-70-10-2012).

### Isolation and Dissociation of CPs for Flow Cytometry

Choroid plexuses were isolated from the LVs as described previously (45). They were dissociated in the presence of collagenase type IV (400 U/ml, Worthington Biochemical Corporation, Lakewood, NJ, USA) for 45 min in 37°C, as described in Ref. (13), after which the tissue was mechanically separated and stained with fluorescent antibodies. Single-cell analyses were performed with a 13-channel flow cytometry analyzer (CytoFLEX, configuration B5-R3-V5; Beckman Coulter, Brea, CA, USA). Flow cytometry results were analyzed with FlowJo.

### IP Injection of LPS

Adult C57BL/6 mice were injected IP with 500 ng of LPS (from *E. coli* 055:B5; Sigma-Aldrich, St. Louis, MO, USA) per gram of body weight.

### CP RNA Preparation and qPCR

Mice were perfused with PBS, and CPs were isolated following *in vivo* experiments or collected 4 h following *ex vivo* coculture experiments with T cells, as described earlier. RNA was extracted from the CPs by using the miRNeasy Micro Kit (QIAGEN, The Netherlands). RNA quality and quantity were examined by NanoDrop 1000 spectrophotometer (Thermo Fisher Scientific Inc., Waltham, MA, USA). The RNA was reverse-transcribed by using a high-capacity cDNA reverse transcription kit (Thermo Fisher Scientific Inc., Waltham, MA, USA). For the quantitative real-time PCR analysis, 20 ng cDNA was used per well. All genes were analyzed with the TaqMan Gene Expression Assay using commercial probes (Thermo Fisher Scientific Inc., Waltham, MA, USA). The GAPDH gene, showing stable expression under the different treatments we used and throughout the replication cycles, was used as an endogenous control to normalize gene expression.

### Isolation, Activation, and Polarization of Polyclonal CD45.1^+^CD4 T Cells

Splenocytes from CD45.1 C57BL/6 mice were harvested and the CD4 T cells were separated by using the EasySep mouse CD4 T-cell negative enrichment kit (StemCell Technologies, Canada). Enriched polyclonal CD4 T-cells were then activated with anti-CD3/anti-CD28 DynaBeads (Thermo Fisher Scientific Inc., Waltham, MA, USA) in a complete DMEM medium (10% fetal calf serum, 10 mM HEPES, 1 mM sodium pyruvate, 10 mM non-essential amino acids, 1% Pen/Strep/Nystatin, and 50 µM β-mercaptoethanol). A Th1 polarization cocktail (which includes 20 µg/ml of anti-mouse IL4 and 1 ng/ml recombinant IL-12; BioLegend, San Diego, CA, USA) was also added during activation. Twenty-four hours after the activation, the T cells were detached from the beads, washed, and resuspended in PBS for experimental use.

### Intracerebroventricular Injection of CD4 T Cells

Mice were anesthetized with isoflurane and the CD4 T cells were injected either at a resting state or 24 h following activation with anti-CD3/anti-CD28 DynaBeads (Thermo Fisher Scientific Inc., Waltham, MA, USA). Before injecting resting OVA-specific T-cell lines, the live cells were purified from the dead cells and debris by using Lympholyte-M (Cedarlane lab, Canada). In some experiments, the injected T cells were pre-labeled with the green cell proliferation tracer CFSE (Thermo Fisher Scientific Inc., Waltham, MA, USA). The cells were then resuspended in PBS at a concentration of 100,000 cells/μl, and a total of 2.5 × 10^5^ cells were injected with a stereotactic device into each of the LVs of the brain [coordinates relative to the bregma: latero-lateral (*x*) =+1/−1, dorso-ventral (*y*) = −0.5, rostro-caudal (*z*) = −2.30] at a rate of 1 µl/min.

### Intrathecal Injection Into the Cisterna Magna

Mice were injected IP with 400 µl of 20% mannitol (B. Braun Medical Inc., Germany) 20 min before the ICM injection. A 25-gauge needle, curved at a 45° angle, 2.5 mm from the tip, was used for the ICM injection, as described in Ref. (46). Each mouse was ICM-injected with 20 µl of solution containing 500 ng of recombinant IFN-γ (BioLegend, San Diego, CA, USA) and either PBS, 1 µg of an OVA_323–339_ peptide (GenScript Corp., Piscataway, NJ, USA), or 1 µg of an MOG_35–55_ peptide (GenScript Corp., Piscataway, NJ, USA).

### Resting OVA-Specific Th1 Cell Line

Splenocytes derived from OT-II TCR Tg mice were cultured with 10 µg/ml of OVA_323–339_ (GenScript Corp., Piscataway, NJ, USA) and stimulated in the presence of recombinant murine IL-2 (20 U/ml; PeproTech, Rocky Hill, NJ, USA) in complete DMEM medium. One week after the first activation, and every 2 weeks thereafter, the cells were stimulated with irradiated splenocytes (6,000 rad) and 10 µg/ml of the OVA_323–339_ peptide. In the first three stimulations, a Th1 polarization cocktail (which includes 20 µg/ml of anti-mouse IL4 and 1 ng/ml recombinant IL-12; BioLegend, San Diego, CA, USA) was also added during activation. The cells were collected for ICV injection during their non-proliferative resting state (confirmed by the lack of ELISA-cytokine secretion, proliferation, and a low-level expression of activation markers identified by flow cytometry), 3 weeks after their last activation.

### Immunohistochemistry

Mice were euthanized with an overdose of isoflurane and perfused with cold PBS. Their brains were immersed in a 4% paraformaldehyde solution at 4°C overnight, transferred to a 30% sucrose solution at 4°C for 2 days, and fixed in OCT (Tissue-Tek, Torrance, CA, USA). Sagittal sections (40 µm) of the brain were produced with a cryostat and kept at −20°C. The sections were rinsed twice in a washing solution (0.05% PBS/Tween 20) and permeabilized for 15 min in 0.5% PBS/Triton X-100. Before staining, the sections were subjected to a primary antibody diluting buffer (Biomeda, Foster City, CA, USA) for 30 min to block non-specific binding. Fluorescently stained sections were examined under an Olympus FV1000 laser-scanning 4-channel confocal microscope (Olympus, Hamburg, Germany).

### Confocal Image Analysis

Confocal images were generated with a 4-channel OLYMPUS XI81-ZDC confocal microscope. Images were acquired based on setting the laser’s sensitivity with negative control sections as well as with sections stained only with secondary antibodies.

#### Three-Dimensional Reconstruction

For 3D images, a z-stack of at least 25 µm thickness, with serial images taken every 0.75 µm, was imaged in the confocal microscope.

#### Measurement of ICAM-1 Intensity

Quantification analysis of ICAM-1 on the epithelial cells of the CP was performed in three sections (each 35 µm thick) per hemisphere immunolabeled for ICAM-1. In each section, the LV CPs were imaged for quantification with the confocal microscope. Fluorescent intensity was measured in all sections by using identical laser-scanning parameters for the entire experiment. Using the Imaris™ image analysis software (Bitplane, Zurich, Switzerland), an intensity threshold was set to mark only those areas that showed significant staining. The sum of fluorescent intensity was calculated for each LV CP and normalized to the recorded volume of that CP. All results were standardized to the control treatment.

#### Measurement of CFSE and Ki-67 Expression in CD4^+^ T Cells

Quantification analysis was performed in three sections (35 µm thick) per hemisphere immunolabeled for CD4 and Ki-67. The injected cells were pre-labeled with CFSE. In each section, the LV CPs were imaged for quantification with the confocal microscope. Fluorescent intensity was measured in all sections by using identical laser-scanning parameters for the entire experiment. Using the “surface” option in Imaris™, CD4^+^ T cells were defined and separated manually if needed. Next, the CFSE and Ki-67 fluorescent values of each T cell were obtained.

#### Live Imaging Videos Recording

Choroid plexuses were cocultured with CD4 T cells, as described earlier. Frames were captured every 10 min for 12 h.

### Antibodies

#### Immunohistochemistry

Rabbit anti-Claudin-1 (CLD-1, 1:100) was purchased from Proteintech (Chicago, IL, USA); Armenian hamster anti-CD54 (ICAM-1, 1:100) was purchased from BD biosciences (Franklin Lane, NJ, USA); rabbit anti-Iba-1 (1:1,000) was purchased from WAKO (Osaka, Japan); rat anti-I-A/I-E (MHC II, 1:100) and rat anti-CD4 (1:100) were purchased from BioLegend (San Diego, CA, USA); rabbit anti-Ki-67 (1:250) was purchased from Cell Marque (Rocklin, CA, USA); Armenian hamster anti-CD11c (1:50) was purchased from eBioscience (San Diego, CA, USA); goat anti-CD106 (VCAM-1, 1:100) and goat anti-CD31 (PECAM-1, 1:100) were purchased from R&D systems (Minneapolis, MN, USA). Anti-laminin (1:50) was purchased from Sigma-Aldrich (St. Louis, MO, USA); TO-PRO-3 (Invitrogen) and DAPI (Thermo Fisher Scientific Inc., Waltham, MA, USA) were used for counterstaining. Alexa 488, 546, or 633 antibodies (Invitrogen), diluted 1:250–1:500, were used for secondary staining.

#### Flow Cytometry

Anti-CX_3_CR1 (PE), anti-CD11c (PE-dazzle 594), anti-CD45.1 (APC), anti-I-A/I-E (MHC II, AF 700), anti-CD45.2 (APC-cy7), anti-CD11b (BV 421), anti-CD4 (BV 510), anti-CD11b (BV605), anti-CD45.2 (BV650), and anti-CX_3_CR1 (BV785) were purchased from BioLegend (San Diego, CA, USA). Anti-Claudin-1 (AF 488) and its corresponding rabbit IgG isotype control (AF 488) were purchased from Bioss (Woburn, MA, USA). Anti-CD54 (ICAM-1, PE-vio-770) and its corresponding REA isotype control (PE-vio-770) were purchased from Miltenyi Biotech (Germany).

#### Neutralizing Experiment

LEAF purified anti-mouse CD54 (ICAM-1) and LEAF purified rat IgG2b, κ isotype control, were purchased from BioLegend (San Diego, CA, USA).

### Artificial CSF Preparation and *Ex Vivo* Coculturing of CPs and CD4 T Cells

The aCSF that was used in this experiment comprised NaCl (120 mM), NaHCO_3_ (26 mM), KCl (2.5 mM), NaH_2_PO_4_ (1.25 mM), MgSO_4_ (1.3 mM), CaCl_2_ (2 mM), and glucose (10 mM). During preparation, a mixture of O_2_ (95%) and CO_2_ (5%) was bubbled into the aCSF for 20 min. Intact non-perfused CPs were isolated from untreated C57/BL6 mice and from mice expressing GFP under the UBC or Cx3cr1 promoter, incubated in the aCSF at 37°C with O_2_ (95%) and CO_2_ (5%), and cocultured with either non-activated or activated CD45.1^+^ Th1 cells stained with CFSE or SNARF-1 (Thermo Fisher Scientific Inc., Waltham, MA, USA). After 24 h, the CPs were collected from the plate and underwent two vigorous washes to preserve T cells that either firmly adhered to the CP or had transmigrated into the stroma. The washed CPs were dissociated and immunolabeled for flow cytometry.

### Chemokine Receptor Signaling Blockage by PTX

Activated Th1 cells were incubated for 2 h at 37°C in a complete DMEM medium and 200 ng/ml PTX, as described previously (47). After incubation, the cells were washed twice and collected for experimental use.

### Statistical Analyses

We used GraphPad Prism™ for statistical analyses (v5.03) and for generating the heat maps of gene expression (v7.01). To compare between two groups, we used a two-tailed unpaired *t* test. To compare between more than two groups, we used a one- or two-way ANOVA with Tukey’s multiple comparison test or with Bonferroni *post hoc* test, respectively. We used Pearson’s correlation test to correlate between two parameters.

## Ethics Statement

This study was carried out in accordance with the recommendations of the 1994 law for the prevention of cruelty to animals (experiments on animals). The protocol was approved by the university committee for the ethical care and use of animals in experiments. Authorization number: IL-70-10-2012.

## Author Contributions

IS designed and performed the research, analyzed the data, and wrote the manuscript. YE and OB performed the research. JR wrote the manuscript. KM and AN provided technical support for the experiments. AM designed the research and wrote the manuscript.

## Conflict of Interest Statement

The authors declare that the research was conducted in the absence of any commercial or financial relationships that could be construed as a potential conflict of interest.

## Supplementary Material

Video S1 Prolonged interactions between T cells and choroid plexus (CP) epiplexus cells at the apical surface of the CP. Intact CPs (green) were isolated from untreated Cx3cr1^GFP/WT^ mice and cocultured with activated SNARF-1^+^ labeled Th1 cells (red) for live-cell imaging. Z-stack images were taken every 10 min, transmitted light was recorded (grey). Time lapse of a 3.5 h measurement shows continuous interactions between CX_3_CR1^+^ cells and SNARF-1^+^ cells at the apical surface of the CP.

## Abbreviations

aCSF, artificial CSF; APCs, antigen-presenting cells; BBB, blood–brain barrier; BCSFB, blood–CSF barrier; BLMB, blood–leptomeningeal barrier; CFSE, carboxyfluorescein succinimidyl ester; CNS, central nervous system; CP, choroid plexus; CSF, cerebrospinal fluid; DCs, dendritic cells; dpi, days post-injection; EAE, experimental autoimmune encephalomyelitis; ICAM-1, intercellular adhesion molecule 1; ICM, intra-cisterna magna; ICV, intracerebroventricularly; IFN-γ, interferon gamma; IHC, immunohistochemistry; IP, intraperitoneal; LPS, lipopolysaccharide; LV, lateral ventricle; MFI, median fluorescent intensity; MOG, myelin oligodendrocyte glycoprotein; OVA, ovalbumin; qPCR, quantitative PCR; TNF, tumor necrosis factor; VCAM-1, vascular cell adhesion molecule 1.
