# Supplementary material for: The Choroid Plexus Functions as a Niche for T-Cell Stimulation Within the Central Nervous System
Source: Front Immunol. 2018 May 16;9:1066. doi: 10.3389/fimmu.2018.01066 (PMC5962702; doi:10.3389/fimmu.2018.01066)
Supplement: Supplementary file 2 [file data_sheet_1.PDF]

Supplementary Material

The Choroid Plexus Functions as a Niche for T-Cell Stimulation within the Central Nervous System

Itai Strominger, Yehezqel Elyahu, Omer Berner, Jensen Reckhow, Kritika Mittal, Anna Nemirovsky, and Alon Monsonego*

* Correspondence should be addressed to Prof. Alon Monsonego,
E mail: alonmon@bgu.ac.il, Tel: 972-8-6479052, FAX: 972-8-6479051

Supplementary figures

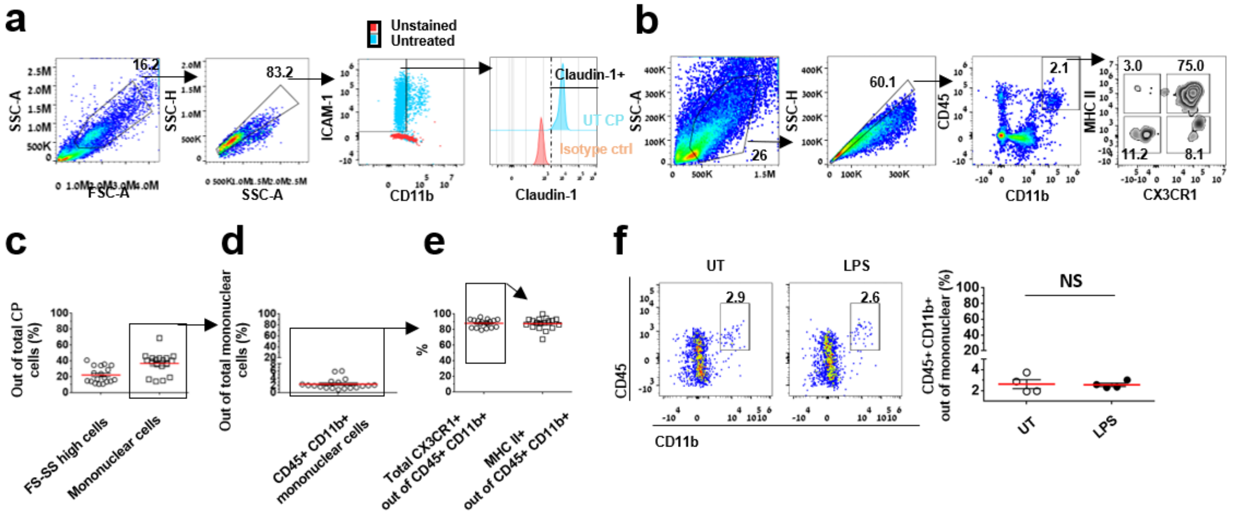

Figure S1

Supplementary Figure 1. Flow cytometry analyses of CP epithelial and myeloid cell subsets. Flow cytometry analysis of adult male C57BL/6 mice (n = 14) were perfused with PBS and their CPs were isolated from the LVs and processed for flow cytometry. The CPs primarily show two distinct populations: CD11b–ICAM-1+Claudin-1+ epithelial cells (**a, c)** and CD45+CD11b+ myeloid mononuclear cells **(b, c).** Analyses of CP mononuclear cells reveal that CD45+CD11b+ cells comprise 1.8% ± 0.8% of the mononuclear cells in the CP **(d)**; of these, 88.2% ± 5.1% are CX_3CR1+ cells, comprising mainly (87.7% ± 7.3%) MHCII+ cells **(e)**. The frequency of CD45+CD11b+ cells in the CP does not change 24 h following an IP injection of LPS (n = 4), as compared with untreated mice (n = 4). Each symbol represents the LV CP from one individual mouse. Bars represent means ± SEM. NS, not significant (P > 0.05; **f**– unpaired *t* test).

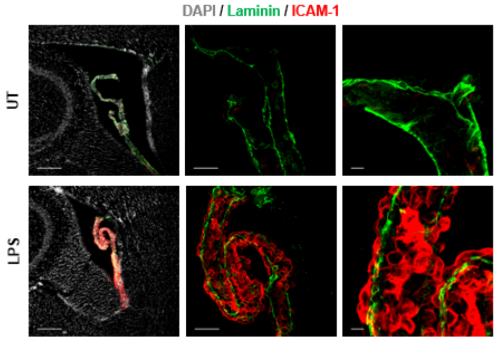

Figure S2

Supplementary Figure 2. Spatial ICAM-1 expression in the LV CP. Adult male C57BL/6 mice were injected intraperitoneally with LPS or kept untreated (UT). Twenty-four hours later, the mice were perfused with PBS and their brains were collected for IHC analyses. Brain sections were immunolabeled with anti-laminin (green) and anti-ICAM-1 (red). DAPI was used for nuclear counterstaining (gray). Confocal images of the LV CPs demonstrate upregulation of ICAM-1 in the CP of LPS-injected mice, colocalized primarily with the CP epithelium but not with CP capillaries. Scale bars represent 200 µm (left panels), 50 µm (middle panels), and 20 µm (right panels).

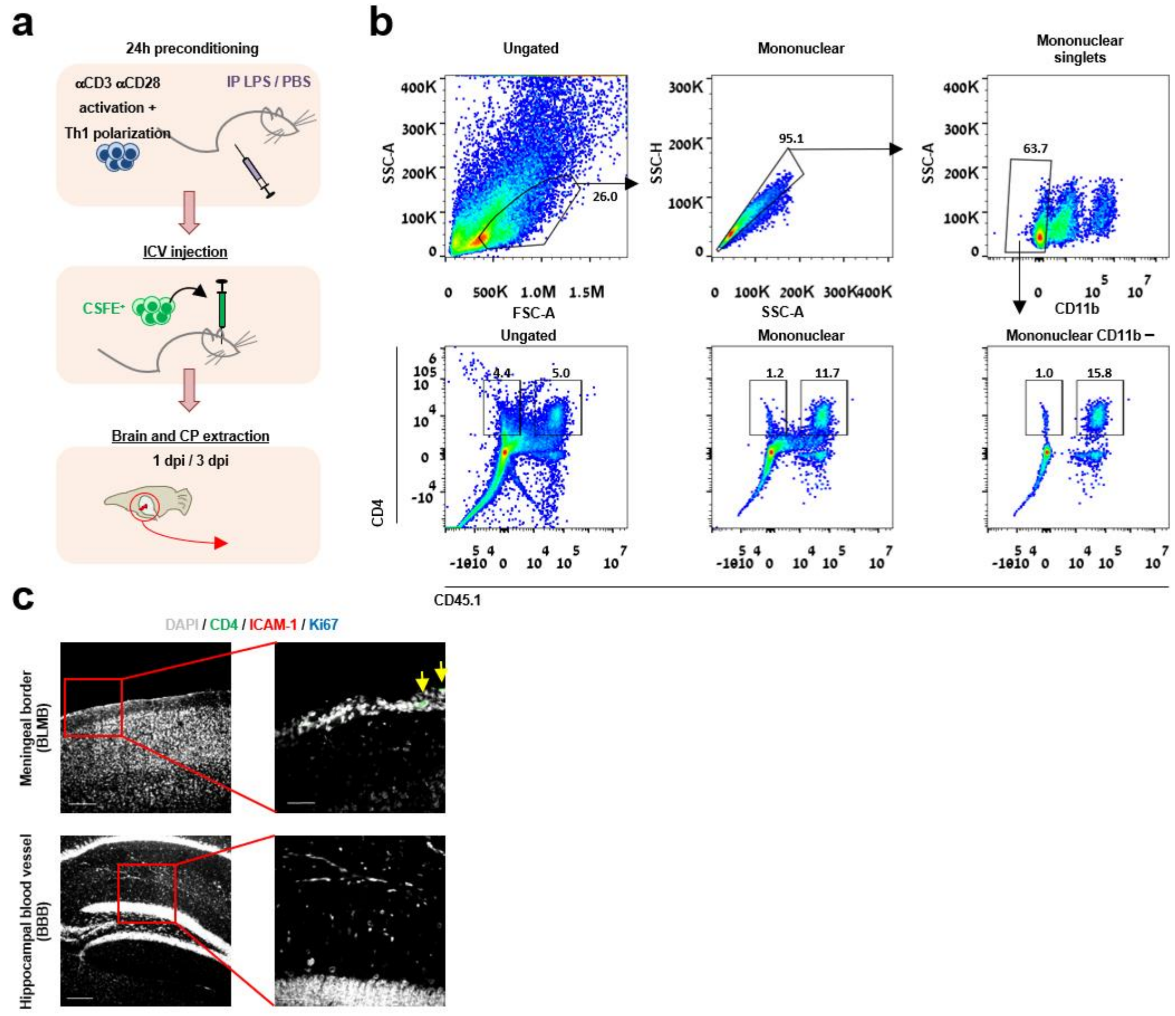

Figure S3

Supplementary Figure 3. Experimental scheme of an ICV injection of activated Th1 cells and their detection in the CNS. (a) CD4 T cells were purified from CD45.1+ splenocytes, activated with anti-CD3/anti-CD28 beads in the presence of a Th1 polarization cocktail for 24 h, and then labeled with CFSE. The CFSE+ T cells were ICV-injected into the LV of mice injected IP with either PBS or LPS, 24 h prior to the injection of the activated Th1 cells. Finally, the CPs were extracted for further analysis, 1 or 3 dpi. **(b)** A flow cytometry gating strategy to identify CD45.1+ injected cells among CP cells. Large population of auto-fluorescent cells make it difficult to distinguish CD45+ leukocytes in their FSC-SSC gate. However, back-gating enabled us to detect CD45.1+ cells as negative in the CD11b BV421 channel, while the auto-fluorescent population appears with a low expression of CD11b (showing the same auto-fluorescent pattern in unstained samples of CP; data not shown). The final gating strategy that identifies CD45.1+CD4+ T cells is indicated by the arrows. **(c)** Representative IHC images, showing the meningeal border of the cortex (representing the blood-leptomeningeal barrier; BLMB; top panels) and parenchymal

blood vessels (representing the blood-brain barrier; BBB; bottom panels) in brain sections of LPS-preconditioned mice, three days following the injection of the activated Th1 cells. Tissues were immunolabeled with anti-CD4 (green), anti-ICAM-1 (red), anti-Ki-67 (blue), and a DAPI nucleus counterstain (gray). Yellow arrows indicate a few CD4 T cells that were identified in the BLMB. Scale bars represent 200 µm (left panels) and 50 µm (right panels).

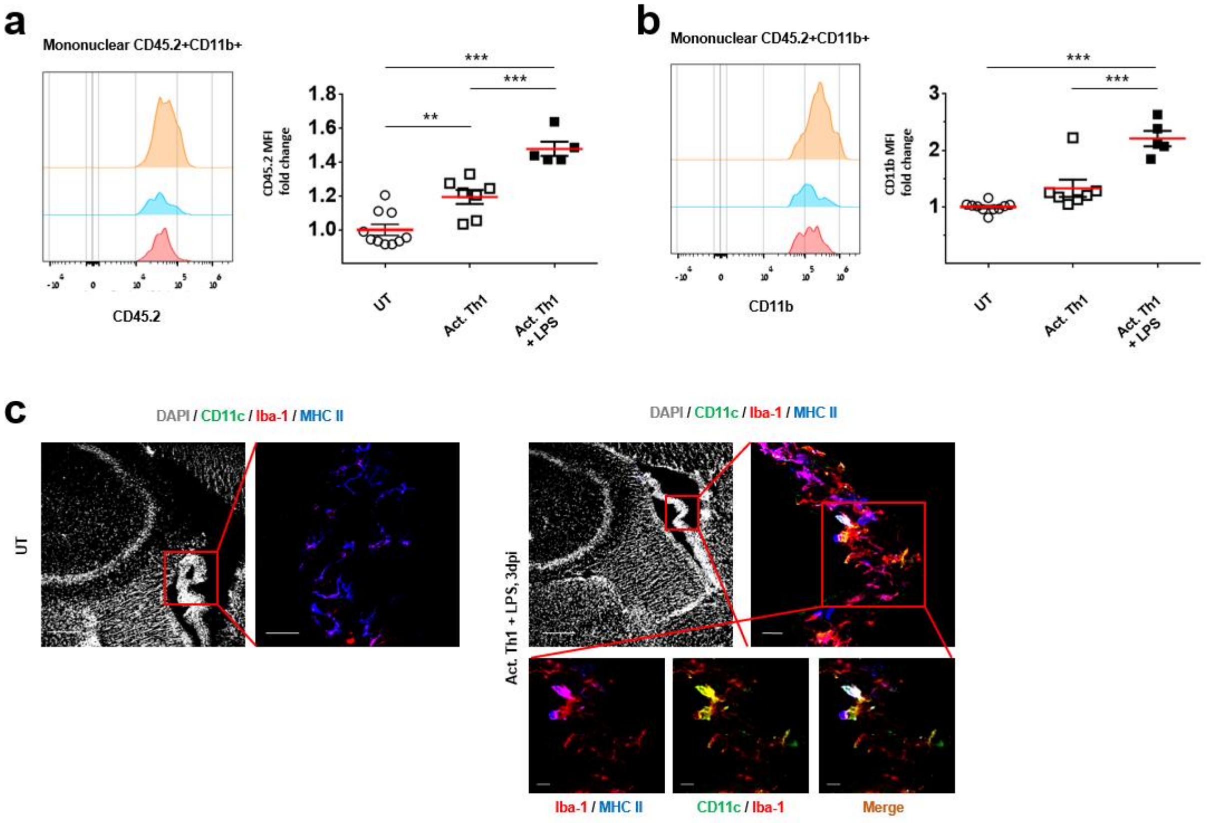

Figure S4

Supplementary Figure 4. Activation of CP myeloid cells following LPS preconditioning and an ICV injection of activated Th1 cells. The flow cytometry analyses of gated CD45.2+CD11b+ mononuclear cells demonstrate higher levels of CD45.2 **(a)** and CD11b **(b)** following an ICV injection of activated Th1 cells to untreated mice (n = 7) which is further increased in LPS-preconditioned mice (n = 5), as compared with untreated (UT) mice (n = 10), measured by median fluorescent intensity (MFI). Each symbol represents one LV CP from an individual mouse. Bars represent means ± SEM. ** $P < 0.01$, *** $P < 0.001$ (one-way ANOVA). Representative brain sections were immunolabeled with anti-CD11c (green), anti-Iba-1 (red), anti-MHCII (blue), and a DAPI nucleus counterstain (gray). IHC images of LV CPs show an increased expression of Iba-1 and CD11c on MHCII+ cells in mice that had been ICV-injected with activated Th1 cells and preconditioned with LPS, as compared with UT mice **(c)**. Scale bars represent 200 µm (top left and middle left panels), 50 µm (top right panels), 20 µm (middle right panels), and 10 µm (bottom panels).

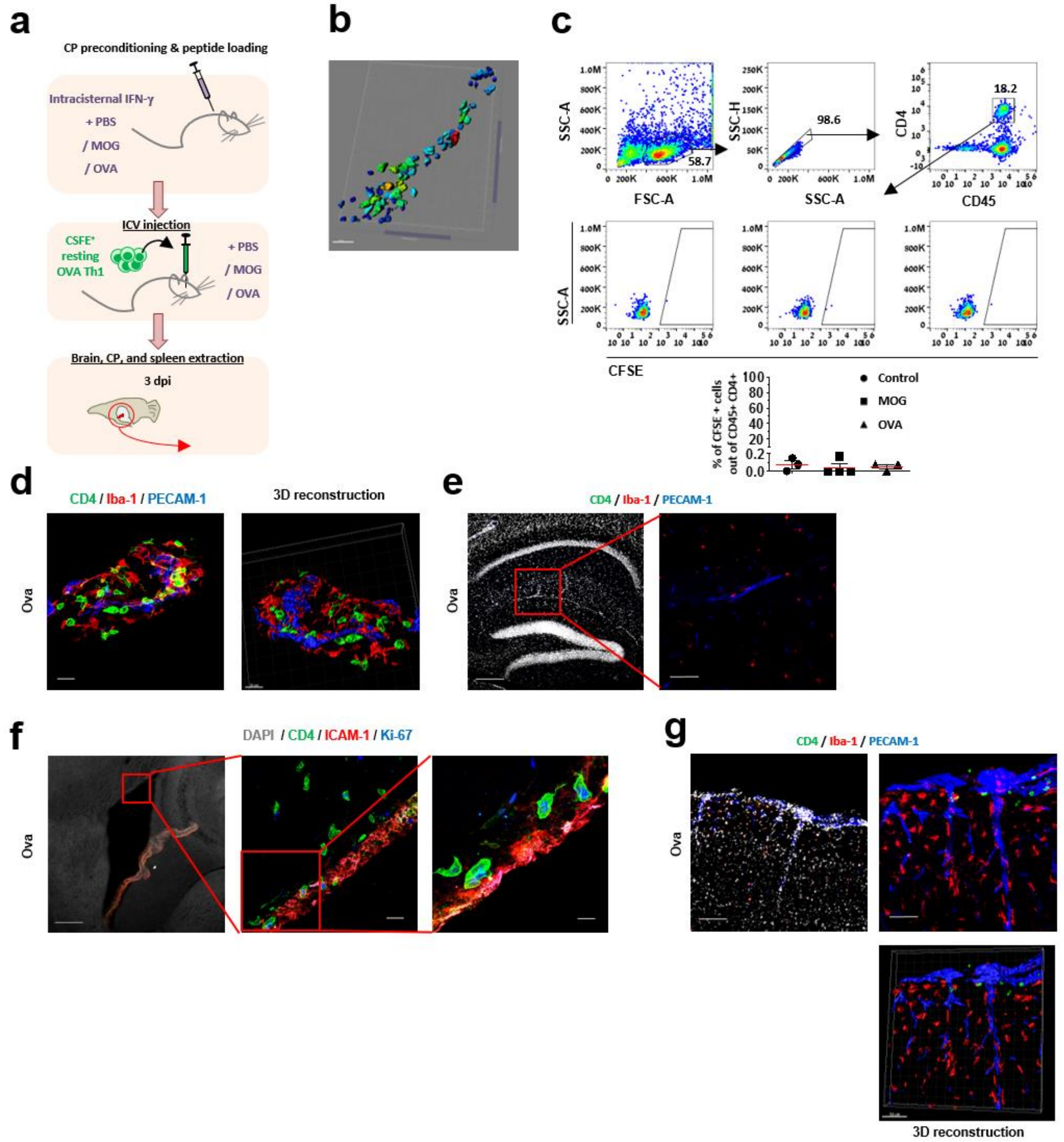

Figure S5

Supplementary Figure 5. ICV-injected T cells undergo antigen-specific stimulation within the CP and transmigrate into the brain parenchyma. (a) C57BL/6 mice were injected intrathecally intra-cisterna magna (ICM) with either OVA (n = 4), MOG (n = 4), or PBS (Control; n = 3), together with 500 ng of IFN-γ. Twenty-four hours later, the mice were ICV-injected with CFSE-labeled resting OVA-specific Th1 cells, together with either OVA, MOG, or PBS. At 3 dpi, the mice were perfused with PBS and their brains, CPs (isolated from right hemisphere), and spleens were collected for flow cytometry **(c)** and IHC **(b, d-g)** analyses. **(b)** Brain sections were immunolabeled with anti-CD4 and anti-Ki-67, and confocal images of the LV CPs were obtained.

Using the Imaris^{TM} software, single CD4+ T cells within the CP were observed in 3D reconstructed images to evaluate the expression of Ki-67 and CFSE. Scale bars represent 40 µm. **(c)** A flow cytometry analysis of splenocytes shows no CFSE+ T cells within the spleen in any of the treatments, suggesting that the ICV-injected Th1 cells did not reach the CP from the peripheral blood. **(d, e, g)** Brain sections immunolabeled with anti-CD4 (green), anti-Iba-1 (red), and anti-PECAM-1 (blue)**. (d)** The right panel shows a 3D reconstruction of z-sections (25.9 µm overall, 0.7 µm/slice) of the left panel, from the CP, revealing CD4 T cells (green) interacting with myeloid cells (Iba-1+; red) in the stroma, but not within PECAM-1+ blood vessels. Scale bars represent 20 µm. **(e)** T cells were not detected inside blood vessel in IHC images of the BBB in OVA-injected mice. Scale bars represent 200 µm (left) and 50 µm (right). **(f)** Brain sections were immunolabeled with anti-Ki-67 (red), anti-CD4 (blue), and a DAPI nuclear counterstain (gray). IHC images show activated CD4+ T cells in the ependymal wall of the LV and in the brain parenchyma. Bars represent 200 µm (left panel), 30 µm (middle panel), and 10 µm (right panel). **(g)** CD4+ T cells can be detected, in IHC images (top panels) and in a 3D reconstruction (bottom panel), in the outer cortex border, in proximity to the CSF circulation which continuous with the ventricular system. Scale bars represent 100 µm (top left panel), 50 µm (right panels).

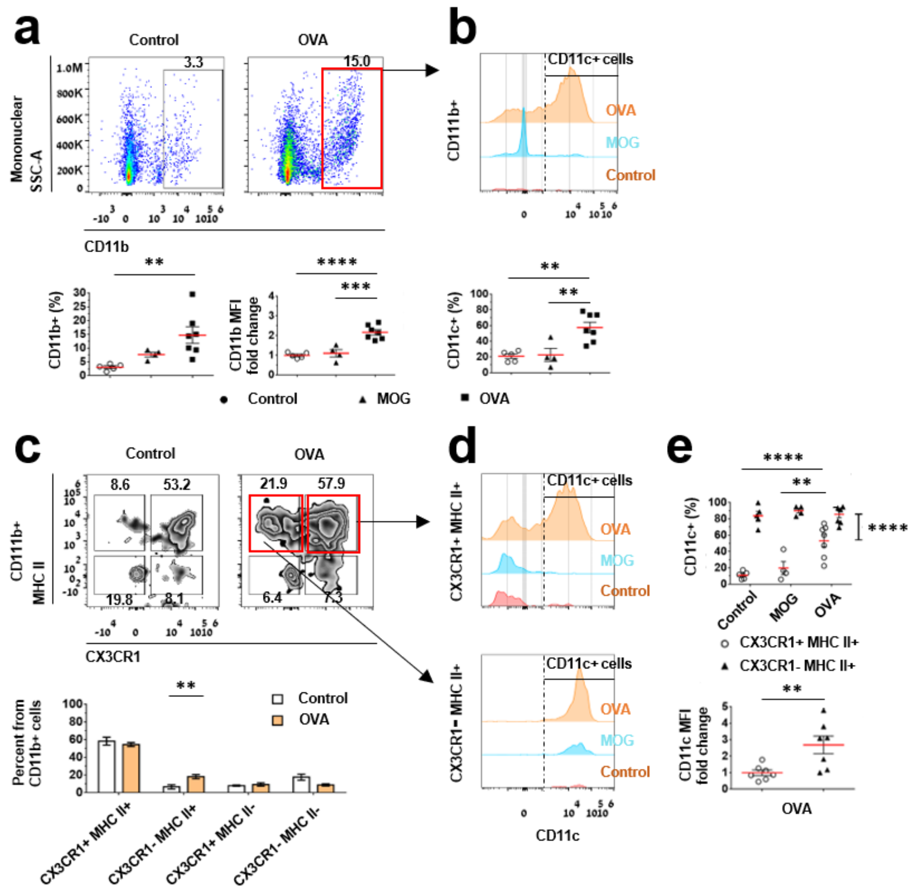

Figure S6

Supplementary Figure 6. Activation of antigen-specific CD4 T cells in the CP promotes the amplification of local antigen-presenting cells. C57BL/6 mice were injected intrathecally intra-cisterna magna (ICM) with either OVA (n = 4), MOG (n = 4), or PBS (Control; n = 3), together with 500 ng of IFN-γ. Twenty-four hours later, the mice were ICV-injected with CFSE-labeled resting OVA-specific Th1 cells, together with either OVA, MOG, or PBS. The CPs were collected 3 dpi for flow cytometry analyses. **(a)** Gated mononuclear cells analysis shows an increased frequency of CD11b+ cells and CD11b expression (measured by MFI) in OVA mice compared with MOG-injected and control mice. The CD11b+ mononuclear cells exhibit an increased frequency of CD11c+ cells **(b). (c)** A flow cytometry analysis of gated CD11b+ mononuclear cells shows an increase in CX_3CR1–MHCII+ cells in OVA mice, as compared with control mice. The frequencies of CD11c+ cells among the CX_3CR1+MHCII+ cells (top) and among the CX_3CR1–MHCII+ cells (bottom) were increased **(d, e-top)**. CP APC populations differ in their CD11c phenotype. In OVA mice, the CX_3CR1–MHCII+ cells show a significantly higher frequency of CD11c+ cells (top) and expression (bottom) of CD11c, as compared with CX_3CR1+MHCII+ cells **(e)**. Each symbol represents a LV CP from one individual mouse. Bars represent means ± SEM.

** P < 0.01, *** P < 0.001, **** P < 0.0001 (**a** and **b** – one-way ANOVA; **c** and **e-top** – two-way ANOVA; **e-bottom** – unpaired *t* test).

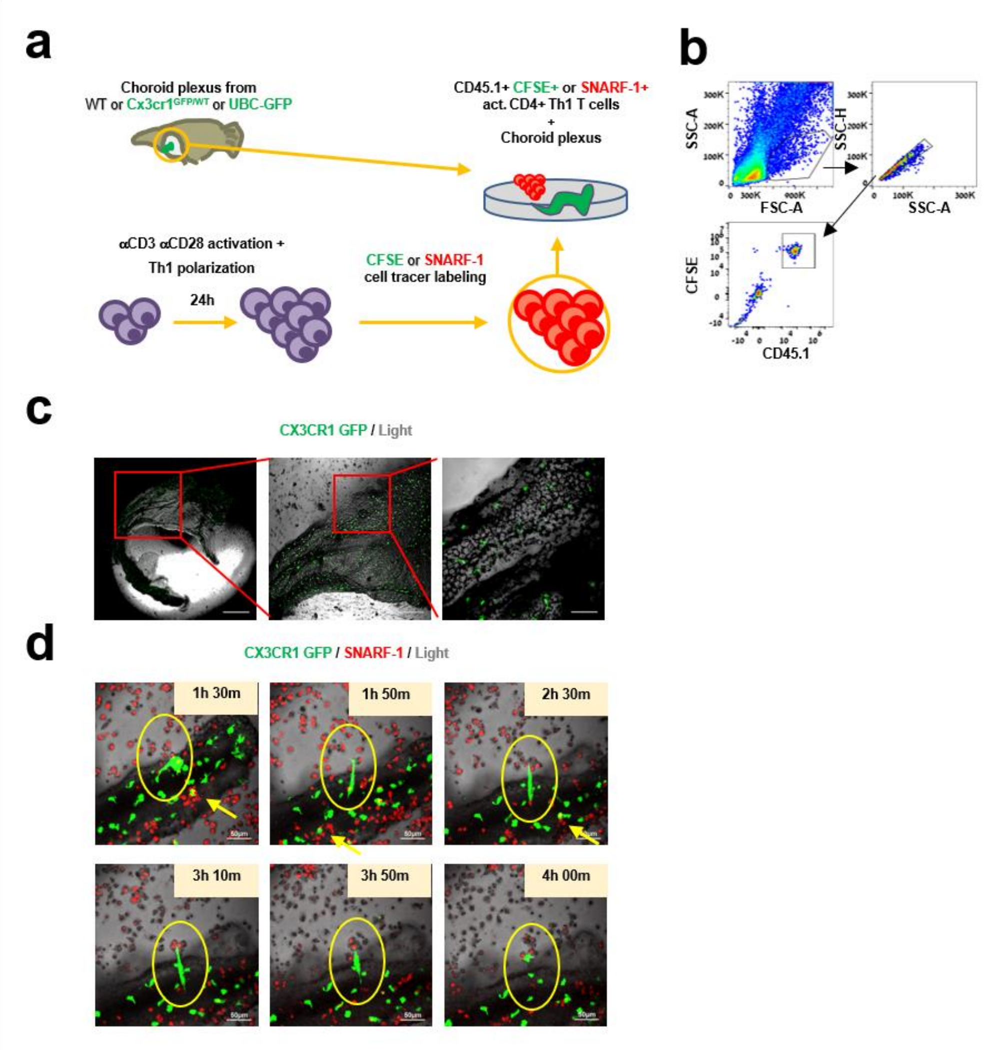

Figure S7

Supplementary Figure 7. ***Ex vivo*** **analysis of T cells homing to the CP and interacting with resident epithelial and myeloid cells. (a)** Brains were excised from non-perfused WT, UBC-GFP, or Cx3cr1$^{GFP/WT}$ mice, and the LV CPs were isolated and cultured in an aCSF. CD4 T cells were purified from CD45.1+ splenocytes by using a negative selection kit, and they were activated with anti-CD3/anti-CD28 in the presence of a Th1 polarization cocktail for 24 h. The T cells were then labeled with CFSE (a green cell tracer) or SNARF-1 (a red cell tracer) and co-cultured with isolated CPs for 24 h. **(b)** Flow cytometry plots show gating strategy to identify the CD45.1+ T cells. **(c)** The distribution of CX_3CR1+ cells in the CPs of untreated Cx3cr1$^{GFP/WT}$ mice. Scale bars represent 500 µm (left panel), 200 µm (middle panel), and 50 µm (right panel). **(d)** Live cell imaging of CPs, co-cultured with SNARF-1+ activated Th1 cells. Confocal time-series images show SNARF-1+ Th1 cells interacting with CX_3CR1+ cells (yellow arrows and circles). Transmitted light was recorded (grey). Scale bars represent 50 µm.

Supplementary tables

Supplementary Table 1a

Gene	Molecular family	LPS 4h (Fold change ± SD)	LPS 24h (Fold change ± SD)
ICAM-1	Adhesion molecules	74.56 ± 35.9	5.93 ± 4.26
VCAM-1		4.19 ± 1.47	2.05 ± 0.67
P-selectin	Selectins	0.48 ± 0.11	1.24 ± 0.6
CD74	Antigen presentation	0.67 ± 0.1	0.48 ± 0.17
CD86	Co-stimulation	4.94 ± 1.58	0.98 ± 0.29
TNF	Cytokines	35.13 ± 6.73	25.41 ± 6.73
IL-1β		12.6 ± 3.89	3.21 ± 1.8
IL-6		1,842 ± 1,171.08	24.02 ± 29.63
IFN-γ		17.21 ± 6.3	1.61 ± 0.62
IL-10		6.99 ± 2.9	3.64 ± 2.36
TGF-β1		1.04 ± 0.25	2.08 ± 0.69
CCL2	Chemokines	277.42 ± 105.72	43.89 ± 17.78
CCL5		63.33 ± 10.47	19.58 ± 8.52
CCL20		402.32 ± 530.53	651.76 ± 562.52
CXCL9		138.98 ± 41.68	6.29 ± 2.59
CXCL10		1,771.49 ± 473.29	54.25 ± 25.85
CXCL11		1,679.12 ± 700.72	49.35 ± 28.42

Supplementary Table 1b

Gene	Statistics (One-way ANOVE)	Tukey's multiple comparison test		
		Control vs. LPS 4h	Control vs. LPS 24h	LPS IP 4h vs. LPS 24h
ICAM-1	$F_{2,14}$ = 24.2, P value < 0.0001	****	NS	****
VCAM-1	$F_{2,14}$ = 16.33, P value = 0.0002	***	NS	**
P-selectin	$F_{2,14}$ = 4.908, P value = 0.0243	NS	NS	*
CD74	$F_{2,14}$ = 8.079, P value = 0.0046	NS	**	NS
CD86	$F_{2,14}$ = 19.74, P value < 0.0001	***	NS	***
TNF	$F_{2,14}$ = 48.38, P value < 0.0001	****	****	*
IL-1β	$F_{2,14}$ = 32.58, P value < 0.0001	****	NS	****
IL-6	$F_{2,14}$ = 15.03, P value = 0.0003	**	NS	***
IFN-γ	$F_{2,14}$ = 38.5, P value < 0.0001	****	NS	****
IL-10	$F_{2,14}$ = 5.212, P value = 0.0203	*	NS	NS
TGF-β1	$F_{2,14}$ = 10.13, P value = 0.0019	NS	**	**
CCL2	$F_{2,14}$ = 34.31, P value < 0.0001	****	NS	****
CCL5	$F_{2,14}$ = 82.88, P value < 0.0001	****	**	****
CCL20	$F_{2,14}$ = 2.863, P value = 0.0907	NS	NS	NS
CXCL9	$F_{2,14}$ = 64.4, P value < 0.0001	****	NS	****
CXCL10	$F_{2,14}$ = 83.12, P value < 0.0001	****	NS	****
CXCL11	$F_{2,14}$ = 34.18, P value < 0.0001	****	NS	****

Supplementary Table 1. A qPCR analysis of CP genes that encode key immune mediators, following a peripheral LPS stimulus. The CPs were excised from the LVs of C57BL/6 mice, either 4 h (n = 5 CPs) or 24 h (n = 7 CPs) after an IP injection LPS or PBS control (n = 5 CPs), and a qPCR analysis was conducted. **(a)** Mean (± SD) fold-change of gene expression following an LPS injection, as compared with PBS control. **(b)** A comparison of the expression of each gene under the various experimental conditions (one-way ANOVA). * P < 0.05, ** P < 0.01, *** P < 0.001, **** P < 0.0001. NS, not significant (P > 0.05).

Supplementary Table 2a

Gene	Molecular family	LPS (Fold change ± SD)	Act. Th1 (Fold change ± SD)	LPS + Act. Th1 (Fold change ± SD)
ICAM-1	Adhesion molecules	0.95 ± 0.14	4.91 ± 2.67	4.58 ± 0.5
VCAM-1		0.97 ± 0.14	2.45 ± 0.87	2.19 ± 0.14
P-selectin	Selectins	2.14 ± 0.86	1.96 ± 0.97	2.43 ± 1.07
CD86	Co-stimulation	1.33 ± 0.42	1.93 ± 0.65	1.81 ± 0.92
TNF	Cytokines	6.98 ± 2.37	8.41 ± 5.15	16 ± 4.44
IFN-γ		1.74 ± 0.86	22.97 ± 19.6	11.76 ± 5.65
CCL2	Chemokines	9.05 ± 3.04	6.32 ± 3.68	18.51 ± 5.83
CCL5		11.13 ± 6.25	12.05 ± 5.49	30.7 ± 21.32
CXCL9		8.77 ± 8.48	1,459.34 ± 2,262.76	2,767.12 ± 1,509.4
CXCL10		6.94 ± 3.8	22.31 ± 9.94	44.4 ± 27.69
CXCL11		13.07 ± 12.81	60.77 ± 32.82	112.39 ± 50.78
CX_3CL1		0.86 ± 0.1	1 ± 0.19	0.81 ± 0.09

Supplementary Table 2b

Gene	Statistics (One-way ANOVE)	Tukey's multiple comparison test					
		PBS vs. LPS + Act. Th1	PBS vs. Act. Th1	PBS vs. LPS	LPS vs. LPS + Act. Th1	LPS vs. Act. Th1	Act. Th1 vs. LPS + Act. Th1
ICAM-1	$F_{3, 15}$ = 11.57, P value = 0.0003	**	**	NS	**	**	NS
VCAM-1	$F_{3, 15}$ = 13.66, P value = 0.0001	**	***	NS	**	***	NS
P-selectin	$F_{3, 15}$ = 2.47, P value = 0.1018	NS	NS	NS	NS	NS	NS
CD86	$F_{3, 15}$ = 2.586, P value = 0.0917	NS	NS	NS	NS	NS	NS
TNF	$F_{3, 15}$ = 12.41, P value = 0.0002	***	*	NS	*	NS	*
IFN-γ	$F_{3, 15}$ = 4.854, P value = 0.0148	NS	*	NS	NS	*	NS
CCL2	$F_{3, 15}$ = 18.22, P value < 0.0001	****	NS	*	**	NS	***
CCL5	$F_{3, 15}$ = 6.051, P value = 0.0066	**	NS	NS	NS	NS	NS
CXCL9	$F_{3, 15}$ = 4.289, P value = 0.0225	*	NS	NS	*	NS	NS
CXCL10	$F_{3, 15}$ = 8.92, P value = 0.0012	**	NS	NS	**	NS	NS
CXCL11	$F_{3, 15}$ = 13.44, P value = 0.0002	***	*	NS	***	NS	NS
CX_3CL1	$F_{3, 15}$ = 3.121, P value = 0.0575	NS	NS	NS	NS	NS	NS

Supplementary Table 2. A qPCR analysis of CP genes that encode key immune mediators after T-cell homing to the CP and a peripheral LPS stimulus. The LV CPs were excised from C57BL/6 mice one day following an ICV injection of activated Th1 cells—either with (1 day before the Th1 cells injection; n = 4 CPs) or without (n = 5 CPs) LPS preconditioning—and of C57BL/6 mice two days following an IP injection of LPS (without the ICV injection of Th1 cells). **(a)** Mean (± SD) fold-change of gene expression, as compared with controls injected ICV with PBS (n = 5 CPs). **(b)** A comparison of the expression of each gene under the various experimental conditions (one-way ANOVA). * P < 0.05, ** P < 0.01, *** P < 0.001, **** P < 0.0001. NS, not significant (P > 0.05).

Supplementary Table 3a

Gene	Molecular family	Non-act. CD4+ (Fold change ± SD)	Act. Th1 (Fold change ± SD)
ICAM-1	Adhesion molecules	1.05 ± 0.3	1.43 ± 0.14
VCAM-1		1.03 ± 0.38	0.5 ± 0.07
P-selectin	Selectins	3.63 ± 0.74	16.8 ± 0.96
CD86	Co-stimulation	0.82 ± 0.11	1.00 ± 0.15
TNF	Cytokines	1.54 ± 0.12	4.46 ± 0.11
IFN-γ		4.34 ± 1.33	69.13 ± 14.68
CCL2	Chemokines	0.96 ± 0.25	0.74 ± 0.22
CCL5		2.73 ± 0.28	2.45 ± 0.23
CXCL9		0.71 ± 0.23	7.29 ± 2.86
CXCL10		1.2 ± 0.24	5.75 ± 0.67
CXCL11		0.95 ± 0.29	5.76 ± 2.28

Supplementary Table 3b

Gene	Statistics (One-way ANOVE)	Tukey's multiple comparison test		
		Control vs. Non-Act. CD4+	Control vs. Act. Th1	Non-Act. CD4+ vs. Act. Th1
ICAM-1	$F_{2, 8}$ = 3.569, P value = 0.078	NS	NS	NS
VCAM-1	$F_{2, 8}$ = 74.41, P value < 0.0001	NS	****	****
P-selectin	$F_{2, 8}$ = 360.7, P value < 0.0001	**	****	****
CD86	$F_{2, 8}$ = 0.3145, P value = 0.7388	NS	NS	NS
TNF	$F_{2, 8}$ = 1017, P value < 0.0001	***	****	****
IFN-γ	$F_{2, 8}$ = 53.99, P value < 0.0001	NS	****	****
CCL2	$F_{2, 8}$ = 0.3353, P value = 0.7247	NS	NS	NS
CCL5	$F_{2, 8}$ = 27.46, P value = 0.0003	***	***	NS
CXCL9	$F_{2, 8}$ = 0.7825, P value = 0.4893	NS	NS	NS
CXCL10	$F_{2, 8}$ = 85.02, P value < 0.0001	NS	****	****
CXCL11	$F_{2, 8}$ = 7.805, P value = 0.0132	NS	*	*

Supplementary Table 3. A qPCR analysis of CP genes that encode key immune mediators, following an *ex vivo* co-culturing of isolated CPs and CD4 T cells. The CPs were excised from the LVs of C57BL/6 mice and were co-cultured either with non-activated CD4 T cells (n = 4) or with activated Th1 cells (n = 4). Four hours later, the total cells in the culture were lysed and the RNA was produced for qPCR analysis. **(a)** Mean (± SD) fold-change of gene expression, as compared with CPs cultured without CD4 T cells (n = 3 CPs). **(b)** A comparison of the expression of each gene under the various experimental conditions (one-way ANOVA). * P < 0.05, ** P < 0.01, *** P < 0.001, **** P < 0.0001. NS, not significant (P > 0.05).

Supplementary Video 1. Prolonged interactions between T cells and CP epiplexus cells at the apical surface of the CP. Intact CPs (green) were isolated from untreated Cx3cr1$^{GFP/WT}$ mice and co-cultured with activated SNARF-1+ labeled Th1 cells (red) for live-cell imaging. Z-stack images were taken every 10 min, transmitted light was recorded (grey). Time-lapse of a 3.5 h measurement shows continuous interactions between CX_3CR1+ cells and SNARF-1+ cells at the apical surface of the CP.
